# Nanosecond Carrier Lifetime of Hexagonal Ge

**DOI:** 10.1021/acsphotonics.4c01135

**Published:** 2024-09-30

**Authors:** Victor T. van Lange, Alain Dijkstra, Elham M. T. Fadaly, Wouter H. J. Peeters, Marvin A. J. van Tilburg, Erik P. A. M. Bakkers, Friedhelm Bechstedt, Jonathan J. Finley, Jos E. M. Haverkort

**Affiliations:** † Eindhoven University of Technology, Department of Applied Physics, Groene Loper 19, Eindhoven, 5612AP, The Netherlands; ‡ Physik Department and Walter-Schottky-Institut, Technische Universität München, Am Coulombwall 4, Garching, D-85748, Germany; ∥ Institut für Festkörpertheorie und -optik, Friedrich-Schiller-Universität Jena, Helmholtzweg 3/5, Jena, D-07743, Germany

**Keywords:** hexagonal Ge, nanowires, radiative lifetime, bandfilling, photoluminescence, oscillator strength, transition matrix element

## Abstract

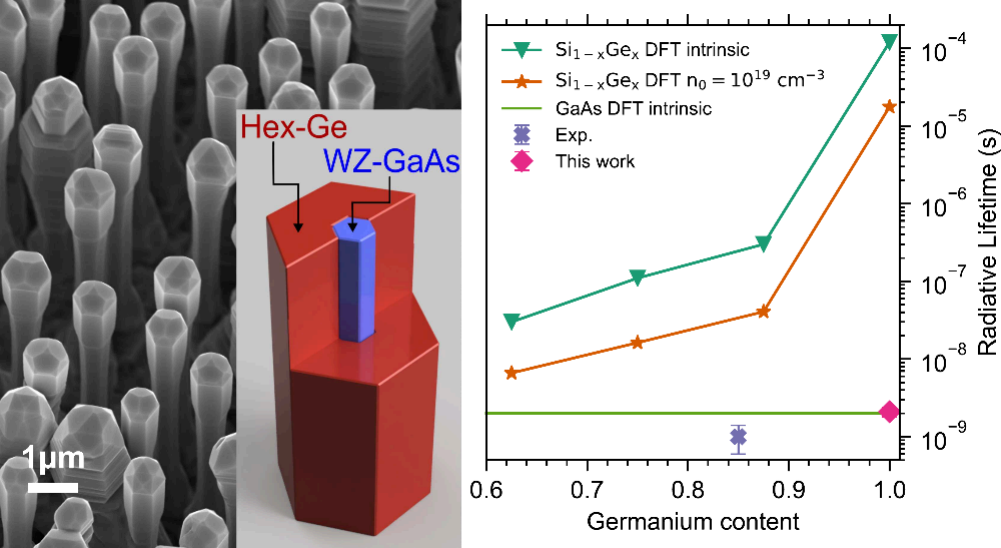

Hexagonal Si_1–*x*_Ge_*x*_ with suitable alloy composition promises to become a new silicon compatible direct bandgap family of semiconductors. Theoretical calculations, however, predict that the binary end point of this family, the bulk hex-Ge crystal, is only weakly dipole active. This is in contrast to hex-Si_1–*x*_Ge_*x*_, where translation symmetry is broken by alloy disorder, permitting efficient light emission. Surprisingly, we observe equally strong radiative recombination in hex-Ge as in hex-Si_1–*x*_Ge_*x*_ nanowires, but scrutinizing experiments on the radiative lifetime and the optical transition matrix element of hex-Ge remain hitherto unexplored. Here, we report an advanced spectral line shape analysis exploiting the Lasher–Stern–Würfel (LSW) model on an excitation density series of hex-Ge nanowire photoluminescence spectra covering 3 orders of magnitude. The analysis was performed at low temperature where radiative recombination is dominant. We analyze the amount of photoinduced bandfilling to obtain direct access to the excited carrier density, which allows to extract a radiative lifetime of (2.1 ± 0.3) ns by equating the carrier generation and recombination rates. In addition, we leveraged the LSW model to independently extract a high oscillator strength of 10.5 ± 0.9, comparable to the oscillator strength of III/V semiconductors like GaAs or GaN, showing that the optical properties of hex-Ge nanostructures are perfectly suited for a wide range of optoelectronic device applications.

## Introduction

The hexagonal crystals of germanium (hex-Ge) and silicon–germanium (hex-SiGe) with the 2H lonsdaleite crystal structure have recently emerged as new direct bandgap semiconductors compatible with silicon.^1−3^ They promise to form the essential building block that was missing in the silicon platform to expand possibilities for the close integration of optical and electronic functionalities.

**Figure 1:**
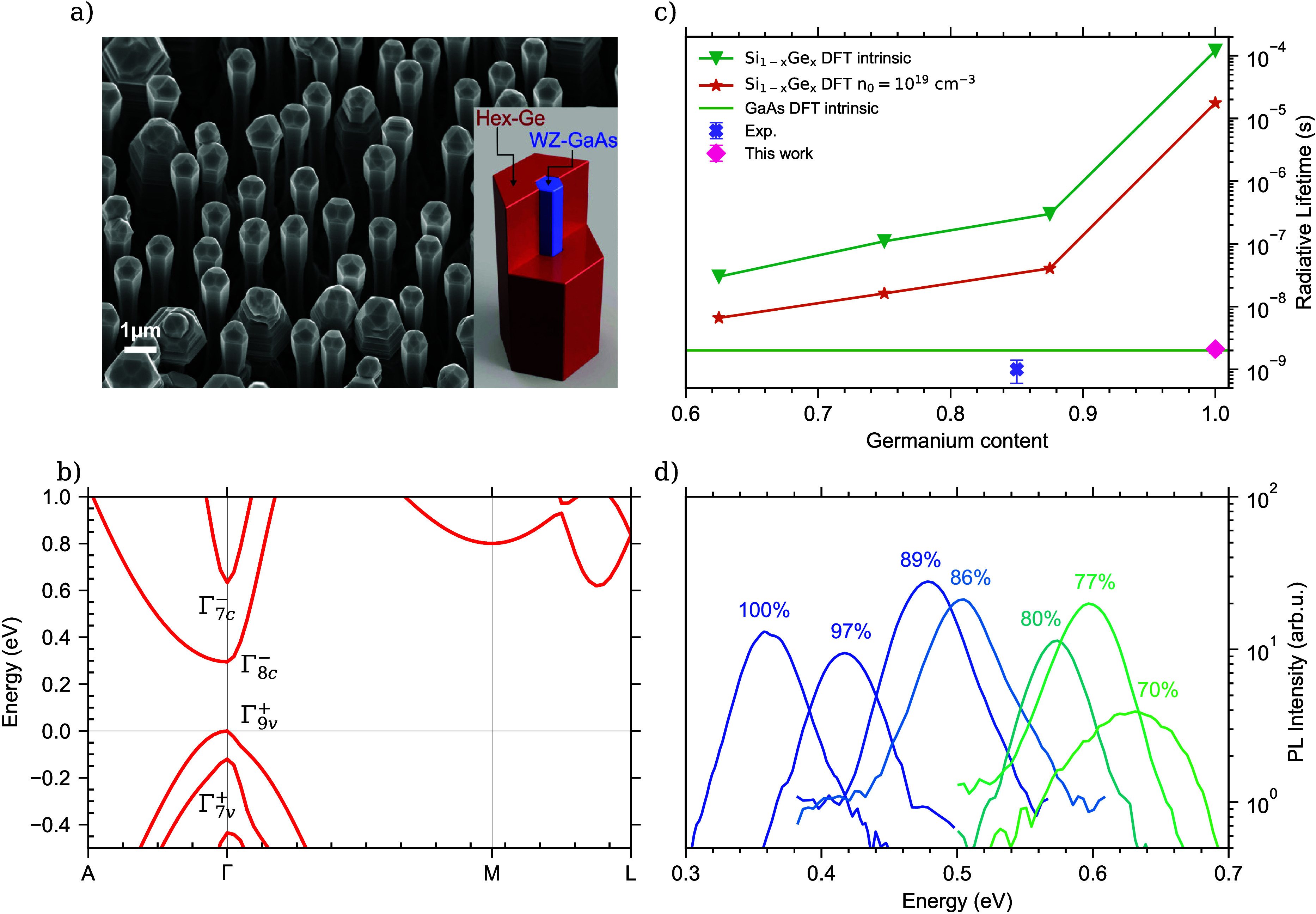
(a) Scanning electron microscope image of the characterized hex-Ge nanowire shell sample. The inset shows a schematic with a partial cross-section of a single core/shell (GaAs/Ge) nanowire. (b) Band structure of the hex-Ge 2H crystal calculated using density functional theory and approximate quasi particle corrections.^4,5^ (c) Theoretically calculated^14^ and experimentally measured,^1^ low temperature, radiative lifetimes of hex-Si_*x*_Ge_1–*x*_ nanowire alloys vs Ge-composition. The radiative lifetime of GaAs is added as a benchmark. The lines connecting the data points only serve as a guide to the eye. (d) The photoluminescence intensity as a function of the Ge-composition showing a virtually constant emission at a temperature of 4 K and at an excitation density of 5.1 kW cm^–2^.^1^ Reproduced and modified with permission from Fadaly et al. *Nature***2020**, *580* (*7802*), 205–209. Copyright 2020 Springer Nature.

Hex-Ge is realized as a nanowire shell as presented in Figure 1a. It has been shown that by tuning the composition of hex-Si_1–*x*_Ge_*x*_, in the range of 0.65 < *x* < 1, direct bandgap band-to-band emission between 1.5 and 3.5 μm can be achieved.^1^ In addition to exhibiting a direct bandgap, the radiative efficiency of this transition is also determined by the transition matrix element, which is composition dependent as well. For instance, Hex-Si_0.2_Ge_0.8_ has been shown^1^ to feature a B-coefficient for radiative recombination similar to that of InP and GaAs. Meanwhile, ab initio Density Functional Theory (DFT) calculations predict that the elemental end point of this family, bulk hex-Ge, is only weakly dipole active due to the Γ_8*c*_^–^ symmetry of the conduction band and would, therefore, exhibit a pseudodirect bandgap.^4−7^ The bandstructure of hex-Ge is shown in Figure 1b. The calculated matrix element of the lowest energy interband transition for bulk hex-Ge crystals provides a radiative lifetime of >0.1 ms, decreasing to 20 μs by adding 10^19^ cm^–3^ n-type doping.^1,4^ The radiative lifetime of bulk hex-Si_1–*x*_Ge_*x*_ crystals is calculated to be significantly shorter^8,9^ with respect to hex-Ge, reaching ≈10 ns, caused by alloy disorder disturbing the lattice symmetry.

Experimental data^1^ from Time-Correlated Single Photon Counting (TCSPC) measurements, however, show a substantially smaller radiative lifetime of around 0.7 ns for hex-Si_0.2_Ge_0.8_. We emphasize that the carrier lifetimes of hex-Ge nanowires are not readily accessible in a photoluminescence lifetime experiment due to the lack of sensitive detectors with picosecond resolution in the wavelength region around 3.5 μm, in part due to the increased blackbody radiation further in the mid-infrared. A comparison of the calculated and measured radiative lifetimes is presented in Figure 1c.

A first indication that hex-Ge features a strongly allowed optical transition is provided by comparing the photoluminescence (PL) intensity^1^ from hex-Ge nanowires at cryogenic temperatures (4K) to that of hex-Si_1–*x*_Ge_*x*_ nanowires, as shown in Figure 1d with an estimated radiative efficiency within 1 order of magnitude compared to III/V materials.^1^ This experimental result suggests a significant discrepancy between the theoretically predicted and experimentally measured optical transition strengths. We note that similar discrepancies have been previously reported for wurtzite III/V semiconductor nanowires.^10−13^

In this work, we focus on an independent determination of both the radiative lifetime and the magnitude of the transition matrix element of hex-Ge nanowire shells. We achieve the hexagonal crystal structure by growing the Ge-shell around wurtzite (WZ) GaAs nanowire cores and thus transferring the hexagonal crystal structure from the hexagonal core to the shell^1^ as shown in Figure 1a. The GaAs core has a diameter of (175 ± 5) nm, and the total nanowire diameter is (0.71 ± 0.03) μm for the thinner middle part and (1.12 ± 0.08) μm for the thicker top, the nanowire length is approximately (6.4 ± 0.4) μm, details are provided in SI I. The shell is thick enough to exclude quantum confinement,^3^ while exciton formation is excluded due to the high n-type doping by arsenic incorporation.^1^

We exploit the lack of alloy broadening due to the elemental nature of hex-Ge, allowing an accurate analysis of the photoluminescence line shape. This is achieved using a generalization of the Planck radiation formula for the blackbody radiation spectrum based on work by Lasher, Stern, and Würfel^15−17^ to model the band-to-band recombination in semiconductors. Hereafter, we will refer to this as the Lasher–Stern–Würfel (LSW) model, as has been recently applied for the extraction of the carrier density by accurate fitting of the photoluminescence line shape in GaAs nanowires,^18,19^ as well as in highly n-type and p-type doped planar GaAs layers.^17,20^

Since the carrier recombination rate *R*_rec_ should be equal to the carrier generation rate *g* under steady-state excitation conditions, which reads as
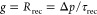
1the extraction of the minority carrier density Δ*p* from the PL line shape provides direct access to the carrier recombination lifetime τ_rec_. For an optically thick sample, the carrier generation rate is directly given by the excitation laser intensity. The final step in our reasoning is that, at low temperature, the carrier recombination lifetime τ_rec_ is equal to the radiative lifetime τ_rad_, i.e., recombination is entirely dominated by radiative processes. We will provide evidence that the sample is in the radiative limit at 4K by comparing hex-Ge to the other hex-SiGe alloys. Independent of the determination of the carrier recombination time, the LSW model additionally provides information on the magnitude of the transition matrix elements, which are expressed in terms of the Kane energy or as a (dimensionless) oscillator strength, as the matrix elements can directly be related to the absorption coefficient at the absorption edge. Moreover, a detailed analysis of the photoluminescence line shape provides a wealth of information on the optical and materials properties of this new hexagonal crystal semiconductor.

## Results and Discussion

### Photoluminescence Spectrum of hex-Ge Nanowire Shells

**Figure 2:**
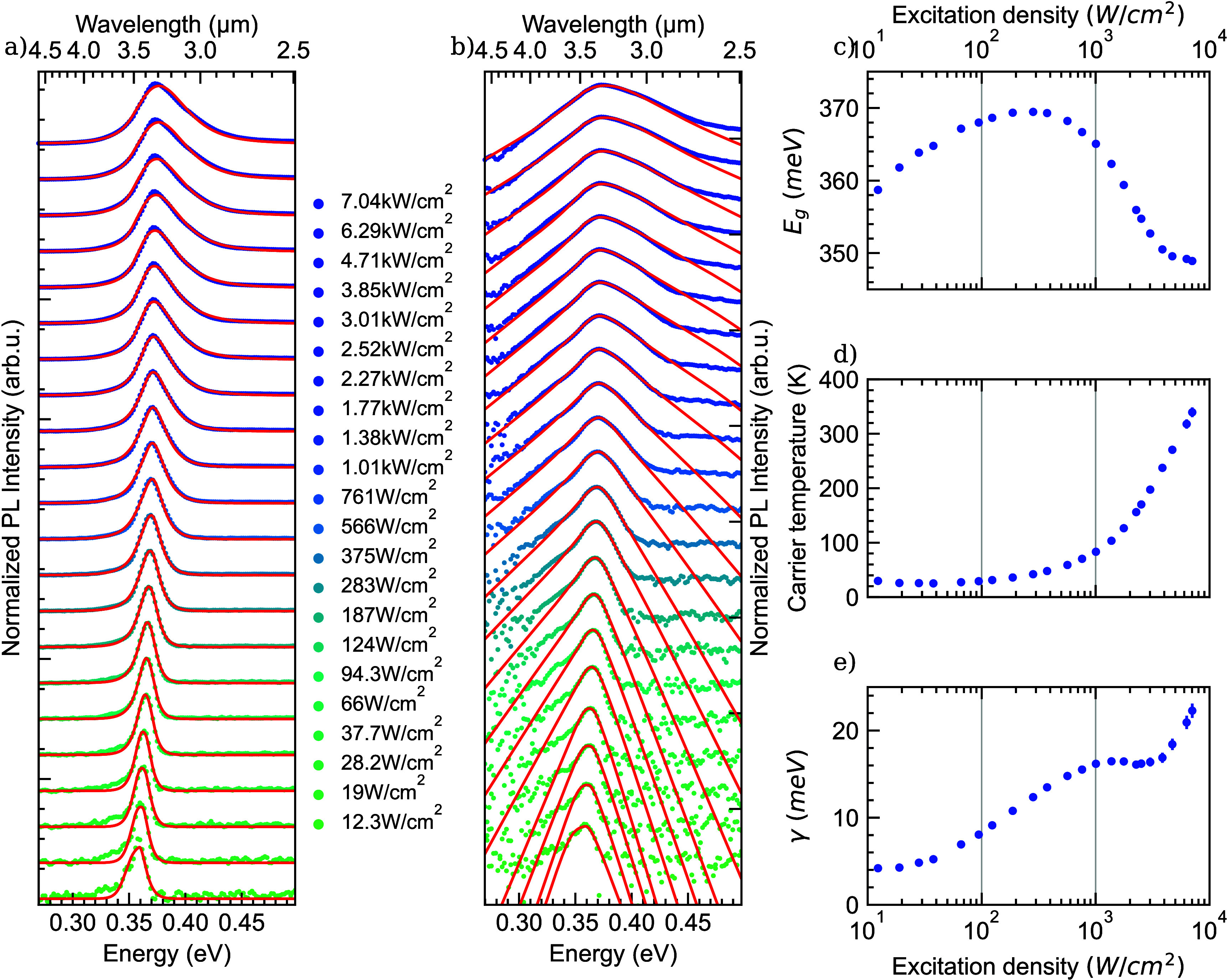
(a) Photoluminescence spectra of the hex-Ge nanowire shells for increasing excitation density (from bottom to top), plotted on a linear scale and fitted with the LSW model. The fits are shown as the red curves. (b) Identical PL spectra as in (a), plotted on a logarithmic scale. (c) Extracted bandgaps from the LSW model for all individual PL spectra shown in (a), displaying the evolution of the bandgap versus excitation density. (d) Evolution of the extracted carrier temperature with excitation. (e) Urbach broadening energy vs excitation.

To accurately model the material properties of hex-Ge nanowire shells, we measured low-temperature photoluminescence spectra using Fourier Transform Infrared Spectroscopy, as further explained in the Methods section. We measured an excitation density series of twenty-two different PL-spectra as presented in Figure 2a on a linear scale, and in Figure 2b on a logarithmic scale in order to be able to accurately extract the amount of Burstein–Moss bandfilling, which in turn determines the total recombination rate. In our LSW analysis, we are not fitting a single PL-spectrum, but we simultaneously fit the complete excitation series of PL spectra with the LSW model, as overlaid in red in Figure 2. The fits show very good agreement, except for the lowest excitation densities where we observe weak and saturable impurity related transitions which are not included in the LSW model. A complete overview of the fitted parameters is given in Table 1. It is important to note that the remarkable close agreement between the fits and the data unambiguously show that the observed PL-spectra features a band-to-band recombination origin.

**Table 1:** Input and Output Parameters of the Lasher–Stern–Wurfel (LSW) Model, Showing the Input Values as Well as the Extracted (Fitted) Output Values^a^

parameter	fit class	input value	extracted output
*E*_g_	dynamic	0.354 eV	0.349–0.369 eV
*T*	dynamic	4 K	25–350 K
γ_c_	dynamic	10 meV	4–22 meV
ζ	stable	≲10^1^ V eV^–2^	(0.23 ± 0.03) V eV^–2^
α_0_*d*	stable	10^–3^–10^3^	(22 ± 1)
*n*_0_	stable	10^19^ cm^–3^	(8.6 ± 0.5) × 10^18^ cm^–3^
Δ*n*/*G*_laser_	stable	10^11^–10^18^ cm^–3^ mW^–1^	(8.2 ± 0.3) × 10^15^ cm^–3^ mW^–1^
θ	constant (literature)	0.5–2.0	1.0
*m*_e_/*m*_0_	constant (theory)	0.179	
*m*_h_/*m*_0_	constant (theory)	0.112	
*G*_laser_	constant (known)		

a Here, *E*_g_ is the hex-Ge bandgap energy, *T* is the carrier temperature, γ_c_ is the Urbach broadening energy of the conduction band edge, ζ is an overall prefactor describing the collection efficiency of our setup, α_0_*d* is the product of the fundamental absorption α_0_ and the sum of the absorption depth and diffusion length (*d* ≤ *L*_NW_), *n*_0_ is the doping density, Δ*n*/*G*_laser_ is the proportionality constant between the excited carrier density Δ*n* and the laser excitation power *G*_laser_, θ describes the shape of the Urbach tails. We take θ = 1.0, which describes semiconductors accurately,^17,19^*m*_e_ and *m*_h_ are the averaged effective masses of the conduction band and valence band, respectively, taken from theory^4^, and *G*_laser_ is the excitation power of the laser. The second column indicates whether the parameter is fitted for each individual PL-spectrum (Dynamic) for the excitation series of the 22 PL-spectra (Stable) or if the parameter is put in as a constant.

### Approaching the Radiative Limit

**Figure 3:**
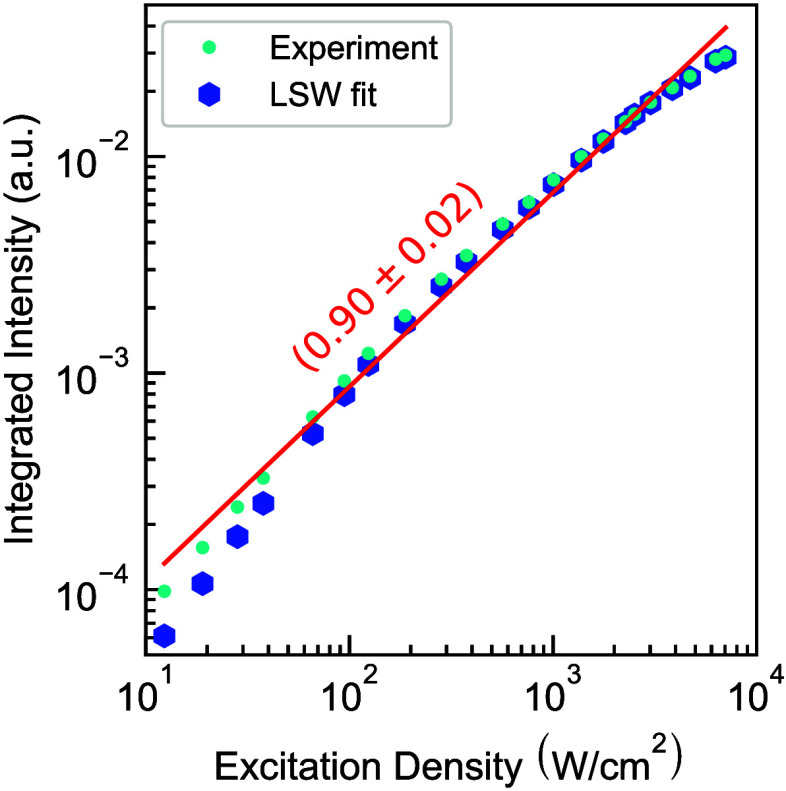
Integrated photoluminescence intensity of hex-Ge nanowire shells as a function of the excitation density at (4 K) for both the experimental data and the LSW fits. The slope of the Light-In–Light-Out curve of 0.90 ± 0.02 is displayed in red and is close to unity, as expected for a material in the radiative limit.

For applying our LSW analysis and extracting the radiative lifetime, it is a prerequisite that the total recombination rate at 4 K is dominated by radiative recombination. We have already shown in the paper by Fadaly et al.^1^ that the nonradiative recombination in hex-Si_0.2_Ge_0.8_ is quenched at 4 K. The fact that the photoluminescence quenching rates in hex-Ge,^1^ which can be expressed as 1/τ_nr_ = (1/τ_nr,0_)*e*^–*E*_act_/*k*_B_*T*^, are even smaller than for hex-Si_0.2_Ge_0.8_ and vanish at low temperature, provide the first compelling argument that nonradiative recombination is quenched at 4 K. Additionally the Light-In–Light-Out (LILO) curve presented in Figure 3 shows a near unity (0.90 ± 0.02) slope over 3 orders of magnitude of excitation density, providing a second argument that hex-Ge is close to the radiative limit at low temperature. A tentative explanation for the slightly sublinear LILO slope is carrier overflow into I3 basal stacking faults^21,22^ as surface recombination of the native hex-(Si)Ge surface was determined not to be limiting the PL.^23^ At the lowest excitation densities, we observe a small contribution from nonradiative Shockley–Read–Hall recombination, while we observe a slightly smaller slope at the highest measured excitation densities, but we emphasize that the deviation from the fit remains smaller than a factor of 2 for all individual data points. Moreover, as hex-Si_0.2_Ge_0.8_ shows pure radiative recombination at low temperature, the comparable magnitude of the PL efficiencies of hex-Ge and hex-Si_0.2_Ge_0.8_ in Figure 1d implies that hex-Ge also closely approaches the radiative limit at low temperature and at excitation densities between 10^2^ and 5.1 × 10^3^ W cm^–2^, as shown in Figure 3. In this work, we will not only extract a nanosecond radiative lifetime, but also a large oscillator strength, providing additional evidence that we approach the radiative limit at 4 K. We finally extract an external radiative efficiency of (57 ± 7) %, which further confirms our analysis, but leaves an error margin of at most a factor of 2 in the radiative lifetime to be extracted in this paper.

### Lasher–Stern–Würfel Model

Our model is based on the standard LSW equation, which describes the photoluminescence spectrum *j*_γ_(ℏω) of a semiconductor in terms of its absorptivity *a*(ℏω) by
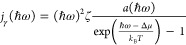
2with the amplitude prefactor ζ = *C*_sig_(4π^2^ ℏ^3^*c*^2^)^−1^, where ℏ is the reduced Planck’s constant, *c* is the speed of light, ℏω is the photon energy, Δμ is the quasi-Fermi level splitting, *k*_B_ is the Boltzmann constant, *T* is the temperature, and *C*_sig_ is a conversion factor that translates the model’s original units into the units of the experimental measurements. For the case of a planar sample, the absorptivity can be expressed in terms of the absorption coefficient α(ℏω) via
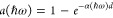
3with *d* being the characteristic length scale over which generation, diffusion, and recombination of charge carriers take place; more details are given in Supporting Information A. This Lasher–Stern–Würfel (LSW) equation shows that in quasi-equilibrium, band-to-band recombination is fully described by the quasi-Fermi level splitting and the absorption coefficient of a material. Assuming a finite, wavevector-independent optical transition matrix element, we subsequently introduce the absorption coefficient α(ℏω), which is given by α(ℏω) = (α_0_/ℏω)(ℏω – *E_g_*)^1/2^, representing the joint density of states (JDOS) and the optical transition strength of a material with a bandgap *E*_g_, where constants are combined into the fundamental absorption strength α_0_. In reality, the absorption edge is not sharp, but broadened by an Urbach tail, which can be described by a convolution of the JDOS with the peak function *U*(*E*, γ, θ) = *N*(γ, θ) exp(−|ℏω/γ|^θ^), in which θ and γ describe the shape and the width of the broadening function respectively and *N* is a normalization constant.^17^ The absorption coefficient, which is accurate for unexcited, intrinsic semiconductors, is then multiplied by the occupation correction factor [*f*_v_ – *f*_c_] ruled by the quasi-Fermi levels μ_e_ and μ_h_ of the electrons and the holes to account for the probability that the initial valence band state is occupied and the final conduction band state is empty, resulting in
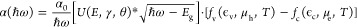
4A detailed derivation of eq 4 together with all required assumptions and a rigorous treatment of α_0_, are given in Supporting Information B and E, respectively.

Equations 2–4 describe a general model for the PL line shape, which was also utilized to describe the PL line shape in planar GaAs, chalcopyrite and kesterite semiconductors, as well as for GaAs nanowires.^17−20^ Further details on the LSW analysis are provided in the Methods section.

### Radiative Lifetime of hex-Ge Nanowire Shells

To determine the radiative recombination lifetime, we require knowledge of the carrier densities in our material. The radiative recombination rate *R*_rad_ is given by
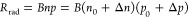
5where *B* is the bimolecular recombination coefficient, and *n* and *p* are the total electron and hole densities, which can be split into the static (doping) densities *n*_0_ and *p*_0_ and the photoexcited carrier densities Δ*n* = Δ*p*. Our investigated hex-Ge nanowires are n-type doped due to the incorporation of arsenic, of the order of *n*_0_ = 9 × 18 cm^–3^, as determined by Atom Probe Tomography (APT) measurements.^1^ The p-type doping due to gallium incorporation is expected to be at least 1 order of magnitude lower. Our LSW fit of the PL line shape yields an activated n-type carrier concentration of *n*_0_ = (8.6 ± 0.5) × 10^18^ cm^–3^, which is perfectly reflected by the APT results that provide the incorporated arsenic concentration. Using the fact that Δ*p* = Δ*n* ≪ *n*_0_ due to the high n-type doping and the moderate CW-excitation, we rewrite eq 5 as
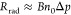
6showing that the radiative recombination rate is determined primarily by the minority carrier density Δ*p*.^24^

As introduced in eq 1, for steady state excitation, *R*_rec_ = *g*, and using a simple rate equation model, the minority carrier lifetime τ_rec_ can directly be extracted from the fitted minority carrier density Δ*p* and the known optical generation rate *g* via
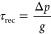
7Considering that the hex-Ge sample is in the radiative limit at 4 K, as reasoned above, we can assume *R*_rec_ = *R*_rad_ = *g* and τ_rec_ = τ_rad_, giving us access to the radiative lifetime. To further improve the accuracy of the extracted lifetime and reduce the number of free parameters, we fit Δ*p*/*G*_laser_ as an overall parameter to the series of all excitation power-dependent PL spectra. This procedure constrains the model to an excitation density independent lifetime, which is a valid assumption when Δ*n* ≪ *n*_0_. We finally obtain
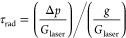
8where *g*/*G*_laser_ is a constant that is derived from known quantities such as the sample geometry (SI I) and the laser excitation density, yielding *g*/*G*_laser_ = (3.8 ± 0.4)×10^24^ cm^–3^ s^–1^ mW^–1^, assuming the laser flux is absorbed completely as we excite high above the bandgap ℏω_laser_ = 1.27 eV ≫ *E*_g_ = 0.35 eV. Using this relation, we determine a radiative lifetime of τ_rad_ = (2.1 ± 0.3) ns at a sample temperature of 4 K. The important result is that the extracted lifetime is 4 orders of magnitude shorter than the theoretical value of 20 μs for ideal hex-Ge crystals.^1^ This implies that the radiative recombination rate is 4 orders of magnitude larger than theoretically expected, which is reflected by the radiative *B* coefficient of *B*_rad_ = 1/(*n*_0_τ_rad_) = (0.54 ± 0.07)×10^–10^ cm^–3^ s^–1^, in which we used the extracted doping density *n*_0_ from the LSW model. Our extracted radiative *B* coefficient is similar in magnitude to the radiative *B* coefficient that we previously established for hex-SiGe.^1^

### Transition Matrix Element of hex-Ge Nanowire Shells

An independent assessment of the transition matrix element is important to elucidate the optical properties of hex-Ge nanowire shells. The strength of the transition matrix element is of particular importance to ascertain that our extracted (2.1 ± 0.3) ns radiative lifetime is due to a high optical transition strength. An independent determination of both a short nanosecond recombination lifetime and a high optical transition strength substantially reinforce our evidence for pure radiative recombination at 4 K, while a low optical transition strength with a short lifetime would indicate nonradiative recombination to be dominant. Notably, the LSW model provides an *independent* method to investigate the optical transition strength by calculating it from the magnitude of the α_0_*d* fitting parameter. The optical matrix elements are usually expressed in the form of the Kane energy *E*_*k*_ for cubic semiconductors or *E*_*k*_⊥__ for hexagonal semiconductors for light polarized perpendicular to the *c*-axis. For hex-Ge, the extracted value of α_0_*d* can be related to the Kane energy by
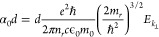
9in which *m*_r_ is the reduced effective mass *m*_r_ = (*m*_e_*m*_h_)/(*m*_e_ + *m*_h_) and *n*_r_ is the refractive index. A full derivation is provided in the Supporting Information E. The fitted value of α_0_*d* = (22 ± 1) eV^1/2^, assuming *d* ≤ (6.4 ± 0.4) μm (limited by the nanowire length) and *n*_r_ ≥ 1 yields a Kane energy *E*_*k*_⊥__ ≥ (3.8 ± 0.3) eV. Although this value is slightly lower than the predicted Kane energy for a wurtzite group III/V semiconductor of 5.27 eV, shown in the Supporting Information E, the Kane energy extracted from the LSW model falls within the order of magnitude expected for a direct bandgap semiconductor. In line with our previously established radiative lifetime for hex-Ge, our extracted value for the Kane energy exceeds the ab initio DFT value taken at the Brillouin zone center of *E*_*k*_⊥DFT__ ≈ 2 meV by 3 orders of magnitude.^4^ For a quantitative comparison with other direct bandgap semiconductors, we also calculate the (dimensionless) optical oscillator strength *f*
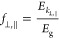
10We find *f*_⊥_ ≥ 10.5 ± 0.9 for hex-Ge nanowire shells, which is slightly lower than the oscillator strength of *f* ≈ 17 for zincblende GaAs, but which is slightly higher than the value of *f* ≈ 5.2 for wurtzite GaN.

Finally, the magnitude of the amplitude prefactor ζ is found to be (0.23 ± 0.03) V eV^−2^ which is lower than the estimated 9.8 V eV^−2^ in (SI G). The difference accounts for additional losses in a real-world optics setup, which might include a yet unknown constant top-facet reflection loss. Replacing the fitting parameter ζ by the original prefactor from eq 2, we arrive at an estimated external radiative efficiency (ERE) of (57 ± 7) %, as shown in more detail in the Supporting Information, Section H. We note that calculations concerning absolute photon counts are nontrivial and the uncertainty in the ERE is likely underestimated. Qualitatively, however, the estimated ERE confirms the main conclusions of the present paper that our hex-Ge nanowire shells combine a nanosecond radiative lifetime with a high oscillator strength, clearly indicating that hex-Ge is a significantly stronger emitter than expected from theory. Moreover, these results provide substantial evidence that hex-Ge nanowire shells, and thus also the hex-Si_1-x_Ge_*x*_ compositional series of semiconductor nanowire shells, are suitable for photonic applications up to the Si-fraction where hex-Si_1-x_Ge_*x*_ turns into an indirect semiconductor.

### Bandgap Renormalization in hex-Ge Nanowire Shells

In the remaining sections, we further investigate the optical properties of our hex-Ge nanowire shells as obtained by the LSW model. Our primary objective is to expand the confidence in the validity of the LSW analysis by showing that we are able to extract physical properties of hex-Ge that are in agreement with standard semiconductor theory. In addition, the LSW analysis allows us to report several yet unknown optical properties of hex-Ge nanowire shells.

**Figure 4:**
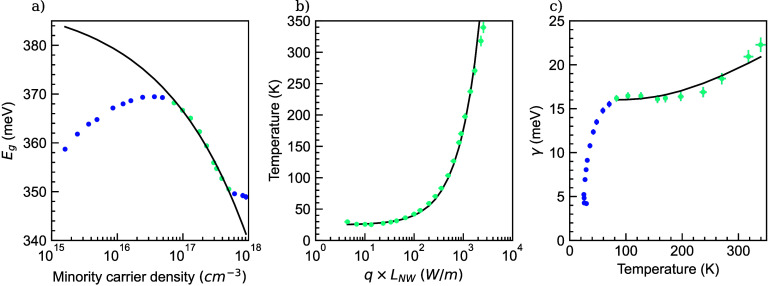
(a) LSW results for the bandgap energy as a function of the minority carrier density. The solid line shows a fit with the Lindefelt model eq 11 to the data where the bandgap is decreasing (cyan markers), showing that the LSW results on hex-Ge can be described with a conventional model for bandgap renormalization in semiconductors. (b) Extracted carrier temperature from the LSW model versus the dissipated power density multiplied with the NW length. The solid line presents a fit with the linear heat transfer formula. (c) Extracted Urbach broadening energy γ vs temperature. The solid line is a fit to the data using eq 12 for temperatures above 77 K (cyan markers), showing that the results on hex-Ge can again be described by conventional theory.

The evolution of the hex-Ge bandgap as a function of excitation density is presented in Figure 2c and clearly shows a high- and low excitation density regime. We attribute the observation of a reduced bandgap at low excitation to excitons or sub-bandgap impurity levels which are beyond the scope of the present paper. At high excitation densities, the extracted bandgap shows a pronounced red shift with increasing excitation density. This observation can be explained by Band Gap Renormalization (BGR) due to the screened exchange interaction.^25^ BGR models have been derived as a function of doping^26^ or photoexcited charge carrier density.^27^ What these models have in common is a ∝ Δ*p*^1/3^ dependence for the shrinkage of the bandgap. Indeed, our data is well explained by fitting expression 11,
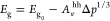
11in which solely a hole–hole self-energy interaction term *A*_*v*_^hh^ is included, to the high excitation part of the bandgap data, as shown in Figure 4a. We mention that, in addition to BGR, an increasing lattice temperature can contribute to bandgap shrinkage. As these contributions cannot be decoupled a more detailed understanding of *A*_*v*_^hh^ is out of the scope of this paper.

### Carrier Temperature in hex-Ge Nanowire Shells

The high energy tail of a PL spectrum is governed by the Fermi–Dirac carrier distribution, which is characterized by the carrier temperature. The steady state carrier temperature can be significantly elevated with respect to the cryostat thermocouple temperature of (4 K) by the excess energy of the 1.27 eV pump laser photons compared to the approximately 0.36 eV bandgap requiring the thermalization of 72% of the incoming power. Moreover, in this nonpolar semiconductor, carrier cooling might be substantially less efficient due to the absence of the efficient Fröhlich electron-polar optical phonon interaction,^28^ and we can therefore also expect a difference in carrier temperature with respect to the lattice temperature of the hex-Ge crystal. The carrier temperature we obtain from the LSW analysis is shown in Figure 2d. From thermodynamics, the carrier temperature is expected to linearly increase with increasing dissipated power. We added the linear increase of the carrier temperature as the solid line in Figure 4b, shown as an exponential curve due to the logarithmic plot. The high quality fit shows that the carrier temperature, extracted from the LSW model, matches the expected linear behavior and further supports the validity of our analysis approach. Importantly, the carrier temperature in our completely nonpolar hex-Ge remains limited to 300 K at high excitation densities up to 7 kW cm^–2^, showing a reasonably efficient carrier cooling in the absence of the Fröhlich interaction.

### Urbach Tail in hex-Ge Nanowire Shells

The evolution of the Urbach broadening energy γ as a function of excitation density is presented in Figure 2e. The Urbach broadening energy increases with higher laser intensity, ranging from 4 to 22 meV and is comparable in magnitude to those in other semiconductors.^29^ Because no theory is available to describe the dependence of γ on excitation density directly, but (empirical) models for the temperature behavior of γ are available,^30^ we have plotted γ versus the temperature, as extracted from the LSW-fit, in Figure 4c. For our interpretation of the behavior of γ, we consider two regimes, either below or above 77 K. In the low temperature regime, γ represents the smearing of the bandgap due to disorder (e.g., stacking faults or charged impurities) in the material while the temperature dependence of γ above 77 K is dictated by the smearing of the bandgap due to local expansions and contractions of the lattice due to phonons. By averaging these lattice vibrations over time, it is clear that the excitation of phonons results in a broadening of the onset of the absorption edge. We fit the Urbach energy as a function of temperature from the start of the plateau (>77 K) toward higher temperatures, using relation 12, which was first introduced by Kurik^31^ as
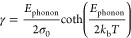
12The resulting fit is shown by the solid line in Figure 4c. The fit of the Urbach broadening energy versus temperature yields a phonon energy *E*_phonon_ of 59 ± 4 meV, which is close to the phonon energy of 37.2 meV obtained by Raman spectroscopy on hex-Ge by de Matteis et al.^32^ In addition, we obtain a value for σ_0_ = 1.8 ± 0.1, which is inversely proportional to the dimensionless exciton–phonon coupling strength.^31^

### Discussion

We report estimates of the radiative lifetime and matrix element where the highest uncertainty, being τ_rad_, is limited to 15%. This is exceedingly accurate to support the 4 orders of magnitude discrepancy with theory. Additionally, we like to stress the mutual consistency of the extracted physical parameters from the LSW model. In particular, the extracted radiative lifetime is consistent with the extracted value of the oscillator strength, and both these quantities are again consistent with the extracted external radiative efficiency. Furthermore, we obtain physically reasonable results for the excitation density dependence of the bandgap renormalization, the dependence of the carrier temperature on excitation density, the extracted activated doping density as well as on the Urbach broadening energy on temperature. Most importantly, the comparable magnitude of integrated photoluminescence intensity of our hex-Ge and the hex-Si_0.2_Ge_0.8_ nanowire shells studied before,^1^ serves as an independent verification of their similarity. The hex-Si_0.2_Ge_0.8_ nanowire shells have already been proven to reach the radiative limit, showing a ≈0.7 ns radiative lifetime.

It presently remains an intriguing question why our experimental results yield a significantly more favorable radiative recombination efficiency than *ab initio* density functional theory for bulk (lonsdaleite, 2H) hex-Ge. We are presently only capable of speculating about the origin of this discrepancy, but we note that similar violations of the predicted optical selection rules for ideal crystals have been observed for wurtzite GaP^12,13^ and the second conduction bands in wurtzite GaAs and InP nanowires.^10,11^

Theoretically, the perturbation due to the hexagonal crystal field is not expected to be strong enough to allow for high oscillator strength at the Brillouin zone center. The most obvious explanation is that translation symmetry is broken by e.g. high doping,^4−6,9,33^ a high stacking fault density^21,22^ or by e.g. the nanowire geometry with real surfaces. Theoretical calculations show that the presence of doping can indeed increase the oscillator strength but this is typically a weak effect.^9^ Alternatively, the high I3 defect density in hex-Si_0.2_Ge_0.8_ nanowire shells clearly destroys translation symmetry and possibly increases the radiative emission efficiency, but this explanation fails to explain the observation of the strong second conduction band Γ_9*v*_^+^ → Γ_8*c*_^–^ transitions in wurtzite InP nanowires.^11^ Another hypothesis is that the optical matrix element increases strongly when moving away from the Γ-point,^4,9,34^ but this explanation implies a reduced PL-efficiency at low excitation, contrary to our observations. We finally note that the application of large, uniaxial tensile strain (≈ 2%) can achieve a Γ_8*c*_^–^ → Γ_7*c*_^–^ conduction band inversion. This inversion is calculated to result in a large optical matrix element and a reduced lifetime, comparable to the observed matrix element and lifetime observed in this work.^5,6,9,33^ Nevertheless, also this explanation is unlikely since strain of that magnitude has *not* been observed experimentally in real nanowire samples.^1^ Moreover, pure hex-Ge is perfectly lattice matched with WZ-GaAs along the *c*-axis, which in combination with the improved strain relaxation due to the nanowire geometry compared to bulk, rules out strain as a cause.

## Conclusions

Since the direct bandgap transition in hex-Ge, Γ_9*v*_^+^ → Γ_8*c*_^–^, is theoretically expected to be very weak, we observe a favorable discrepancy between theory and experiment and provide evidence that this transition is actually strongly allowed in microstructured hex-Ge. The first indication was that the integrated PL-intensity of a hex-Ge nanowire shell is of comparable magnitude compared the PL-intensity of a hex-Si_0.2_Ge_0.8_ nanowire alloy, which are known to feature a short radiative recombination lifetime of 0.7 ns.^1^ Next, we have used an advanced line shape analysis on the photoluminescence spectra of hex-Ge, using a generalization of Planck’s radiation formula known as the Lasher–Stern–Würfel model. We synchronously analyze a series of 22 PL spectra taken from hex-Ge nanowire shells at 4 K, where the excitation density is varied over 3 orders of magnitude. We extract a multitude of physical quantities from the excitation density dependence of the PL-spectra of hex-Ge nanowires. We first focus on the Burstein–Moss bandfilling. Because the amount of bandfilling is inversely proportional to the radiative emission rate, we can extract a radiative lifetime of τ_rad_ = (2.1 ± 0.3) ns. Then, we independently extract the transition matrix element of our hex-Ge nanowire shells from the fit results, yielding an oscillator strength of *f*_⊥_ ≥ 10.5 ± 0.9, similar in magnitude to direct bandgap semiconductors like GaAs or GaN. We emphasize that the combination of a short (2.1 ± 0.3) ns radiative lifetime, a high oscillator strength and a high external radiative efficiency at 4 K, provide compelling arguments that our hex-Ge nanowire shells exhibit comparable optical properties to a III/V semiconductor. We further exploit the LSW model to increase the confidence level of our analysis. We obtain a bandgap renormalization proportional to Δ*p*^1/3^, a linear increasing carrier temperature with excitation power and an Urbach broadening energy providing an approximately correct phonon energy. Since hex-Ge constitutes the origin of the hex-Si_1-x_Ge_*x*_ alloy family, the nanosecond recombination lifetime might further explain the observed nanosecond and subnanosecond recombination lifetimes observed in e.g. hex-Si_0.2_Ge_0.8_, which are also an order of magnitude smaller than the lifetimes predicted by theory. Since we have recently shown direct bandgap emission from hex-Ge/SiGe quantum wells^3^ as well as stimulated emission in single hex-SiGe nanowires,^35^ the experimental evidence reported in this manuscript provides strong evidence that that family of hex-Si_1–*x*_Ge_*x*_ alloys can be further exploited for optoelectronic device applications, including Si-compatible quantum well lasers and quantum dot single photon emitters.

## Methods

### Crystal Growth and FTIR

The growth of the hex-Ge nanowires and the acquisition of its photoluminescence spectra have been discussed in a previous paper.^1^ In short, we have grown wurtzite GaAs nanowire cores which serve to transfer the hexagonal crystal structure toward a hex-Ge shell grown around it. The as-grown sample is mounted in a continuous flow cryostat equipped with a PID-controlled heater which permits is to accurately set the temperature in a range of 2.5 to 300 K. The sample is excited with a 976 nm diode laser with a 45 μm laser spot on the sample, containing approximately 250 hex-Ge wires. We subsequently use a step-scan Fourier Transform Infrared Spectrometer with a Mercury–Cadmium–Telluride (MCT) detector to record the photoluminescence spectrum of our as-grown samples. The laser is modulated at 35 kHz, which allows us to use lock-in technique while the experiment remains to be in quasi-steady-state, because all relaxation processes in the nanowires are orders of magnitude faster.

### LSW Model

To determine the material properties of hex-Ge we leverage the LSW model. The main complexity in the application of the LSW model to describe the PL-line shape arises from the fact that no analytical expressions are available for μ_*e*_ and μ_*h*_ as a function of Δμ. This problem is usually circumvented by using assumptions and/or approximations, among which are the valence and conduction band having a similar effective mass, the semiconductor being intrinsic or low excitation only, see SI, section C. In this work, we choose to only comply with the isotropic parabolic band approximation and use a numerical approach to eliminate all other unknown parameters. We numerically evaluate μ_*e*_ and μ_*h*_ as a function of the photoexcited carrier densities Δ*n* = Δ*p* and doping density *n*_0_, which provides general validity of our model for all temperatures, doping levels and excitation levels, as detailed in Supporting Information D.

A further improvement of the LSW model is to assume that the excited charge carrier density Δ*n* scales proportional to the (known) excitation density (i.e., Δ*n* ∝ *G*_laser_), as described by a proportionality constant Δ*n*/*G*_laser_. The introduction of Δ*n* ∝ *G*_laser_ as a fit parameter reduces the number of fit parameters and increases the accuracy of the obtained lifetime, as it is equivalent to assuming an excitation-density independent lifetime, which is validated by PL lifetime measurements for hex-Si_1–*x*_Ge_*x*_. We finally introduce the fitting variable ζ = *C*_sig_/(4π^2^ℏ^3^*c*^2^) to account for the prefactor in eq 2, where *C*_sig_ describes all experimental losses and conversion factors. Supporting Information G provides an estimate for ζ. A calibration of the absolute spectral irradiance would avoid a fit of ζ and thus eliminate one fitting parameter.^17^ Given the complications associated with measuring the absolute spectral irradiance, we think that a fit of ζ allows for more universal application of the model while retaining the overall accuracy of the fitting procedure by eliminating such a cumbersome absolute calibration.

Although it may look as if our LSW-model requires 11 fit parameters listed in Table 1, the excitation laser power *G*_laser_ is the parameter we vary during the experiment and is therefore known, the effective masses *m*_e_ and *m*_h_ are material constants taken from literature,^4^ θ defines the shape of the Urbach tail and is assumed to 1.0, which describes semiconductors accurately.^17,19^ This leaves seven free parameters for fitting twenty-two spectra. The bandgap energy *E*_g_, the temperature *T* and the Urbach broadening energy γ_c_ are considered dynamic parameters, meaning they are determined for each individual spectrum. We emphasize that these three parameters clearly describe distinctly different features of the PL-spectra, including the spectral position, the line broadening describing the high-energy tail and the line broadening toward low-energy tail. The final four parameters ζ describing the prefactor as mentioned before, α_0_*d* describing the optical absorption strength, *n*_0_ describing the doping density, and Δ*n*/*G*_laser_ being the proportionality constant between the induced carrier density and the laser power are considered (Series-) stable parameters. These parameters can vary, e.g., sample to sample, but are considered stable (quasi-constant) within one full series of PL spectra, resulting in a strong reduction in the degrees of freedom. By fitting complete series, the influence of ζ and α_0_*d* can be easily separated since the height of the PL-spectra scales linearly with ζ, while the height of the PL-spectra scale nonlinearly with α_0_*d*, as given by eq S4.

To fit our numerical LSW model, we make use of a two-step fitting process that combines a standard Levenberg–Marquardt algorithm to fit the dynamic parameters to the individual spectra assuming the stable parameters are constant, with an overarching heuristic iterative Particle-Swarm-Optimization (PSO) algorithm^36^ from the *pyswarms Python* package, which optimizes the stable parameters. PSO is an iterative method where a number of particles are randomly positioned in the problem’s parameter space with a random velocity. In each iteration, every particle is accelerated both down its own gradient and toward the current global best solution. After each iteration, a fitness function is calculated, which is chosen to be the sum of reduced χ^2^ values for all 22 PL-spectra, normalized to the integrated intensity to the power 3/2 to account for the amplitude changes and the signal-to-noise ratio. In this way a large parameter space can be sampled while after a few initial iterations the particles converge toward the best global solution. After this convergence, the particles subsequently investigate the best parameters sets with a higher accuracy. In the second part of the fit, the global parameters obtained from the particle swarm optimization are used as constants to subsequently fit each PL-spectrum using the Levenberg–Marquardt method provided by the *lmfit* package in *Python* to obtain the optimum values for the three parameters which are determined by fits of individual PL-spectra. Here we fit the individual PL-spectra starting with the PL-spectrum obtained at the highest excitation density and sequentially fitting each lower excitation density PL-spectrum in a spectrum-by-spectrum sequence. This approach has the advantage that the PL-spectrum with the highest signal-to-noise level is fitted first. Subsequently, to improve convergence, we use the result of the previous fit as initial conditions for the PL-spectrum currently being fitted. In addition to this sequential fitting procedure, we invoke two additional boundary conditions. Because the lattice temperature was kept constant during the measurement series, it is a reasonable assumption to constrain the fitted temperature *T* of each PL-spectrum to be either equal or decreasing in subsequent fits at decreasing excitation density. Furthermore, the Urbach broadening parameter γ is known to scale with temperature allowing the same constraint.^29^ For computational efficiency we adopt the calculation of the broadened electron–hole interband DOS ρ_JDOS_ from a tabulated generalized DOS ρ_gen_
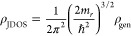
13
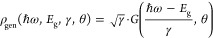
14
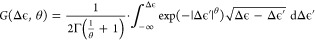
15as suggested by Katahara et al.^17^

Statistical analysis on the PSO iterations show convergence toward a global minimum of the fitness function within the estimated parameter space. An extensive robustness analysis is shown in Supporting Information F.

Knowing the neighborhood of the optimal solution from PSO, we can now leverage this solution as the initial condition for a complete Levenberg–Marquardt-based approach, where the four stable and the three dynamic parameters per spectrum are optimized in one step, to fine-tune the fit and accurately quantify the uncertainties in all parameters.

## Data Availability

The data and code underlying this study are openly available in Zenodo at DOI: 10.5281/zenodo.13358746.

## Supporting Information Available

Extensive derivation and background on the Lasher–Stern–Würfel model focusing on the absorptivity, the absorption coefficient, and the analytic and numerical approach to the occupation correction and quasi-Fermi levels. Derivation of the relation between the absorption strength and the optical matrix element. A robustness analysis of the heuristic Particle-Swarm optimization. An estimation of the amplitude scaling factor ζ. An estimation of the external radiative efficiency of hex-Ge. The sample geometry (PDF)
