# Supplementary material for: Nanosecond Carrier Lifetime of Hexagonal Ge
Source: ACS Photonics. 2024 Sep 30;11(10):4258–67. doi: 10.1021/acsphotonics.4c01135 (PMC11488130; doi:10.1021/acsphotonics.4c01135)
Supplement: Supplementary file 1 — ph4c01135_si_001.pdf [file ph4c01135_si_001.pdf]

Supporting Information

Nanosecond carrier lifetime of hexagonal Ge

Victor T. van Lange,[†,§] Alain Dijkstra,[†,‡,§] Elham M.T. Fadaly,[†] Wouter H.J. Peeters,[†] Marvin A. J. van Tilburg,[†] Erik P.A.M. Bakkers,[†] Friedhelm Bechstedt,[¶] Jonathan J. Finley,[‡] and Jos E.M. Haverkort[*,†]

†*Eindhoven University of Technology, Department of Applied Physics, Groene Loper 19, Eindhoven, 5612AP, The Netherlands*

‡*Physik Department & Walter-Schottky-Institut, Technische Universität München, Am Coulombwall 4, Garching, D-85748, Germany*

¶*Institut für Festkörpertheorie und -optik, Friedrich-Schiller-Universität Jena, Helmholtzweg 3/5, Jena, D-07743, Germany*

§*Contributed equally to this work*

E-mail: j.e.m.haverkort@tue.nl

Number of pages: S26.

Number of figures: 7.

Number of tables: 2.

Supplementary Information

A Description of the absorptivity

To model the absorptance in the Lasher-Stern-Wurfel (LSW) model, we consider the semiconductor as a plan-parallel slab where the diffusion length is much larger than the slab thickness. The distribution of electrons and holes is assumed to be homogeneous and the quasi-Fermi-level splitting to be constant over the slab. The absorptivity of the plan-parallel slab can subsequently be related to the absorption coefficient of the semiconductor, the reflectivities on the front- and back-surfaces $R_f(\hbar\omega)$ and $R_b(\hbar\omega)$ and the slab thickness d_{slab}

$$a(\hbar\omega) = \frac{(1 - R_f)\left(1 - e^{-\alpha d}\right)\left(1 + R_b e^{-\alpha d}\right)}{1 - R_f R_b e^{-2\alpha d}} \tag{S1}$$

as shown by Trupke et al.[1] Alternatively, the d_{gdr} can be defined as the characteristic length scale over which carrier generation, diffusion, and recombination takes place and the aforementioned assumptions stay approximately valid corresponding to the sum of the absorption- and diffusion length of the material.[2] It can be stated with certainty that the effective d can be defined as the minimum of the d_{slab} and d_{gdr}.

Simplifications of this equation can be defined for the special case that $R_b = R_f = R$,[3] yielding

$$a(\hbar\omega) = \frac{(1 - R)\left(1 - e^{-\alpha d}\right)}{1 - R e^{-\alpha d}} \tag{S2}$$

or

$$a(\hbar\omega) = (1 - R_f)\left(1 - e^{-\alpha d}\right) \tag{S3}$$

when the back surface reflection is assumed to be zero ($R_b \approx 0$). This equation can be further simplified if the front surface is assumed to be perfectly anti-reflective $R_f \approx 0$

$$a(\hbar\omega) = 1 - e^{-\alpha d} \tag{S4}$$

which is the equation we choose to use in fitting PL spectra with the LSW model. In the special case that the semiconductor slab is extremely thin $d \to 0$ or excitation of the sample is extremely shallow, for instance with e-beam excitation in cathodoluminesence,[4–6] the absorptivity can be further simplified using a Taylor-series, yielding:

$$a(\hbar\omega) = \alpha d \tag{S5}$$

Here the absorptivity and the absorption coefficient are proportionally related and effects of re-absorption are neglected. Due to the nanowire geometry of our hex-Ge sample, we expect the front-surface reflection to be minimal. Also the back reflection is also assumed to be negligible, due to the small refractive index contrast between hex-Ge $n \approx 3.75$ and the GaAs substrate $n \approx 3.3$. Fortunately, if the front-surface would have a significant influence, an approximate wavelength independent $1 - R_f$ term can simply be contracted into the fitted scaling variable ζ.

B Description of the absorption coefficient model

In Eq. (2) and (3) of the main text, it is shown that the LSW-model directly links the emission properties of a semiconductor to its absorption coefficient. Although most of the equations describing the absorption coefficient are textbook standards, we will shortly summarize the assumptions required to obtain the final model. This is especially important because many physical parameters enter into the fitting model through the absorption coefficient of a two-band semiconductor and the non-equilibrium description by quasi-Fermi levels.

$$\alpha(\hbar\omega) = \frac{\Lambda}{\hbar\omega}\frac{2}{(2\pi)^3}\sum_{\boldsymbol{k}} |\hat{e}\cdot\boldsymbol{p}_{cv}|^2 \delta\left(\epsilon_c(\boldsymbol{k}) - \epsilon_v(\boldsymbol{k}) - \hbar\omega\right) \cdot \left[f_v(\epsilon_v, \mu_h) - f_c(\epsilon_c, \mu_e)\right] \tag{S6}$$

In equation (S6) the most general form of the absorption coefficient[7–11] is presented with the absorption process depicted in figure S1. In this equation,

$$\Lambda = \frac{\pi e^2 \hbar}{n_r c \epsilon_0 m_0^2} \tag{S7}$$

is a material specific constant, specified in SI-units, with e the elementary charge, n_r the refractive index of the material, c the speed of light, and m_0 the rest mass of an electron. Contrary to the usual notation, we take the $1/\hbar\omega$ out of the definition of Λ. This is required since $1/\hbar\omega$ cannot be neglected in the infrared spectral region.

Furthermore $|\hat{e} \cdot \boldsymbol{p}_{cv}|$ is the matrix element describing the probability of a transition from state $|v\rangle$ to $|c\rangle$ (as also illustrated in Fig. S1), in which $\hat{e}$ represents the polarization unit vector and $\boldsymbol{p}_{cv}$ the momentum matrix element. To find all the possible transitions a summation over all $\boldsymbol{k}$-values in the Brillouin zone is carried out in which the Dirac delta function $\delta(\epsilon_c - \epsilon_v - \hbar\omega)$ makes sure that only the transitions that produce a photon with energy $\hbar\omega$ are counted.

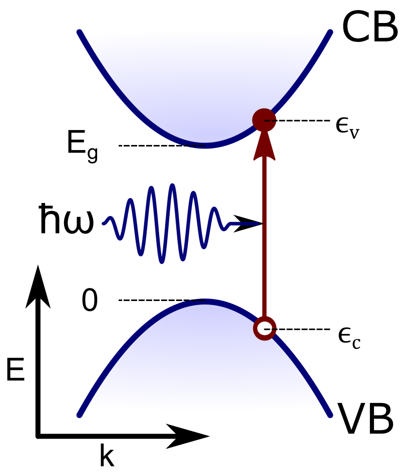

Figure S1: Schematic of the absorption process with all relevant energies marked.

The final factor in Eq. (S6) is the probability that the initial state, with energy ϵ_v, is occupied with an electron and the final state, with energy ϵ_c, is not occupied. The occupation probabilities are included using Fermi-Dirac statistics, both for absorption and stimulated

emission processes, resulting in the relation $f_v\left[1-f_c\right]-f_c\left[1-f_v\right] = f_v - f_c$. Here f_x is the Fermi-Dirac distribution, which can be expressed as $f_x(\epsilon_x, \mu_x) = \left[\exp\left(\frac{\epsilon_x-\mu_x}{k_B T}\right)+1\right]^{-1}$ in which μ_x is the quasi-Fermi-level of the respective charge carriers x.

In equation (S6) it is already implicitly assumed that the transitions are $\boldsymbol{k}$-conserving. Additionally, we will assume that the matrix element $|\hat{e}\cdot\boldsymbol{p}_{cv}|$ is independent of $\boldsymbol{k}$ and can be moved out of the summation. Next, we will presume that both the conduction and the valence band are isotropic and parabolic with their own respective directionally averaged effective masses. The simplified parabolic bands are given by equations (S8) and (S9), in which zero energy is chosen at the valence band edge.

$$\epsilon_v(\boldsymbol{k}) = -\frac{\hbar^2|\boldsymbol{k}|^2}{2m_h} \tag{S8}$$

$$\epsilon_c(\boldsymbol{k}) = E_g + \frac{\hbar^2|\boldsymbol{k}|^2}{2m_e} \tag{S9}$$

The energy of an absorbed photon $\hbar\omega$ must match the energy difference between the valence and conduction band for a given value of $\boldsymbol{k}$, i.e. $\epsilon_c(\boldsymbol{k}) - \epsilon_v(\boldsymbol{k}) = \hbar\omega$. We calculate the transition energy by subtracting Eqs. (S9) and (S8), we obtain a one-to-one relation between the photon energy $\hbar\omega$ and $|\boldsymbol{k}|$, as given in Eq. (S10),

$$\hbar\omega = E_g + \frac{\hbar^2|\boldsymbol{k}|^2}{2m_r}. \tag{S10}$$

Here, the reduced interband mass m_r is introduced which is given by $1/m_r = 1/m_e + 1/m_h$ with m_e and m_h the effective masses of the conduction and valence band, respectively. Due to the parabolic band assumption, Eq. (S6) can be greatly simplified since any value of $\hbar\omega$ now uniquely determines a set of values for ϵ_v, ϵ_c and $|\vec{k}|$. The advantage of this approximation is that the Fermi-Dirac distributions now become independent of $\vec{k}$ and can be moved out of the summation.

The resulting summation over the Dirac delta function is known as the joint density of states $\rho_{JDOS}(\hbar\omega)$, which is the density of pairs of conduction and valence band states which are separated by $\hbar\omega$. Equation (S6) is thus simplified to Eq. (S11).

$$\alpha(\hbar\omega) = \frac{\Lambda}{\hbar\omega}|\hat{e}\cdot\boldsymbol{p}_{cv}|^2\left[f_v(\epsilon_v(\hbar\omega),\mu_h) - f_c(\epsilon_c(\hbar\omega),\mu_e)\right]\rho_{JDOS}(\hbar\omega) \tag{S11}$$

The occupation correction term $f_v - f_c$ in Eq. (S11) needs to be carefully evaluated when the material is highly excited and/or degenerately doped, which will be discussed separately in SI C and SI D. For now we will assume that the semiconductor is intrinsically doped and only lightly excited which means that the occupation correction term reduces to $f_v - f_c = 1$.

In the parabolic band approximation, the joint density of states can be written as[12]

$$\rho_{JDOS}(\hbar\omega) = \frac{1}{2\pi^2}\left(\frac{2m_r}{\hbar^2}\right)^{\frac{3}{2}}\sqrt{\hbar\omega - E_g}\cdot H(\hbar\omega - E_g) \tag{S12}$$

where H is the Heaviside step function. By inserting this into Eq. (S11) in combination with $f_v - f_c = 1$, the well known textbook[13,14] expression for the absorption coefficient for a direct band gap semiconductor is obtained, as expressed by Eq. (S13).

$$\alpha(\hbar\omega) \propto \frac{1}{\hbar\omega}\sqrt{\hbar\omega - E_g} \tag{S13}$$

Unfortunately, the model presented in Eq. (S13) is often insufficient to accurately describe the absorption coefficient. Because of lattice vibrations, the band edges are slightly smeared out, which is known as the Urbach-tail.[15] Other factors contributing to the Urbach tail are impurities and crystal defects.

To incorporate broadening in our model, we choose to convolute the ideal joint density of states $\rho_{JDOS,ideal}(\hbar\omega)$ with an exponential peak function $U(\epsilon)$ resulting in Eq. (S14).

$$\rho_{JDOS}(\hbar\omega) = \int_{-\infty}^{\infty} U(\epsilon')\rho_{JDOS,ideal}(\hbar\omega - \epsilon')d\epsilon' \tag{S14}$$

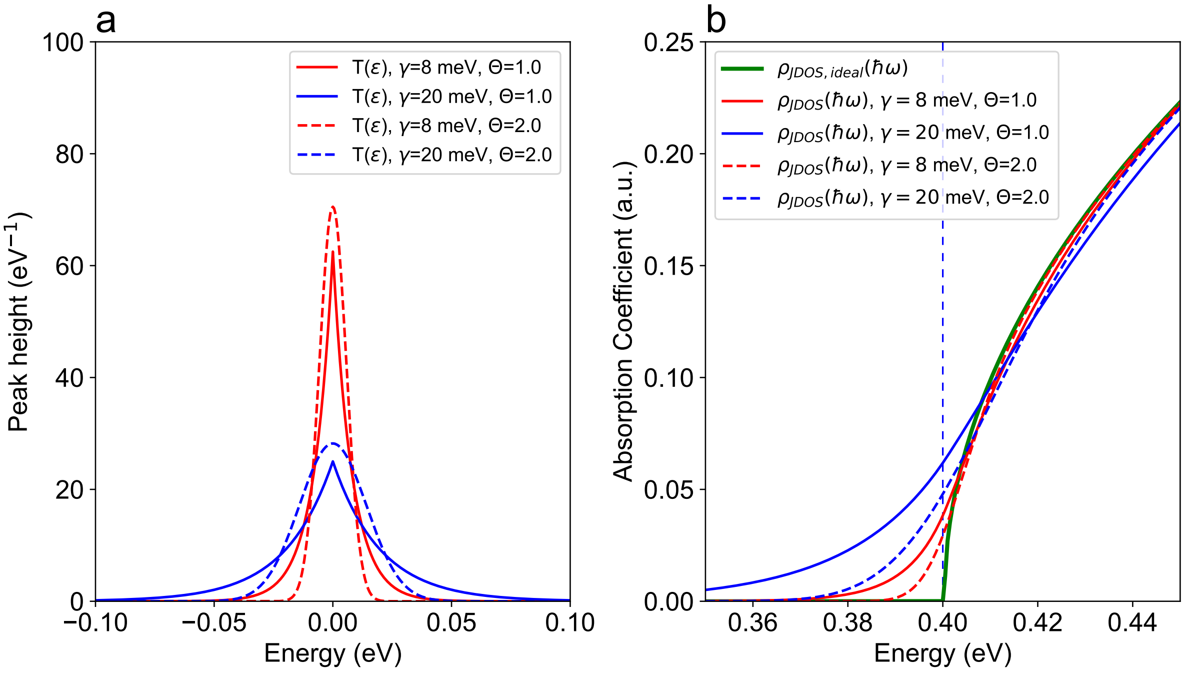

Figure S2: (a) Example broadening (peak) functions $U(\epsilon)$ plotted for two values of θ and two different values of the broadening parameter γ. The resulting broadened absorption coefficients $\alpha_0(\hbar\omega)$ calculated using Eq. (S15) are plotted in (b). The unbroadened $\alpha_{ideal}(\hbar\omega)$ is shown as a reference. The band gap of 0.4 eV was chosen arbitrarily.

For the exponential peak function we use the generalised model suggested by Katahara[2] et al., which is expressed as $U(\hbar\omega) = N(\gamma, \theta) \exp\left(-\left|\frac{\hbar\omega}{\gamma}\right|^\theta\right)$. Here, γ is the broadening parameter and θ is a shape parameter with a value in between 0.5 and 2 depending on the physical origin of the broadening mechanism. Additionally $N(\gamma, \theta)$ is a normalisation constant, given by $[2\gamma \cdot \Gamma(1 + 1/\theta)]^{-1}$, in which Γ is the gamma function which serves to keep the integral of the peak function equal to unity. Combining this peak function with Eqs. (S13),(S14) and replacing the upper limit of the integration to only keep the real part of the integral, we find a complete model for the broadened joint density of states given by Eq. (S15).

$$\rho_{JDOS}(\hbar\omega) = \int_{-\infty}^{\hbar\omega - E_g} N(\gamma, \theta) \exp\left(-\left|\frac{\epsilon'}{\gamma}\right|^\theta\right) \sqrt{(\hbar\omega - \epsilon') - E_g} \quad d\epsilon' \tag{S15}$$

In literature there is no single most appropriate choice for θ, a clear discussion on this problem was published by Katahara[2] et al. As an example, broadening functions $U(\hbar\omega)$ have been plotted in Fig. S2a for two different values of γ and θ, where in S2b the resulting broadened $\rho_{JDOS}(\hbar\omega)$ functions are plotted based on Eq. (S15).

By combining equation (S11) with expression (S15) and by absorbing all constants into a single variable α_0, we find an expression for the absorption coefficient $\alpha(\hbar\omega)$, which reads as (S16)

$$\alpha(\hbar\omega) = \frac{\alpha_0}{\hbar\omega}\left[N(\gamma,\theta)\exp\left(-\left|\frac{\hbar\omega}{\gamma}\right|^{\theta}\right) * \sqrt{\hbar\omega - E_g}\right] \cdot [f_v(\epsilon_v,\mu_h) - f_c(\epsilon_c,\mu_e)] \tag{S16}$$

, in which $*$ indicates a convolution.

C Analytic models of the occupation correction term

The definition of the occupation correction term $[f_v(\epsilon_v,\mu_h) - f_c(\epsilon_c,\mu_e)]$ is given by Eq. (S17).

$$[f_v - f_c] = \frac{1}{\exp\left(\frac{\epsilon_v-\mu_h}{k_BT}\right)+1} - \frac{1}{\exp\left(\frac{\epsilon_c-\mu_e}{k_BT}\right)+1} \tag{S17}$$

Here ϵ_v (ϵ_c) is the energy of the hole (electron) in the valence band (conduction band) that takes part in the recombination process, and μ_h (μ_e) the quasi-Fermi level of the holes (electrons). In order to make this expression compatible with the LSW model the variables ϵ_v, ϵ_c, μ_h and μ_e must be expressed in terms of $\Delta\mu$, E_g and $\hbar\omega$. For ϵ_c and ϵ_v this is straight forward under the parabolic bands assumption, by combining equations (S8), (S9) and (S10), Eqs. (S18) and (S19) are found.

$$\epsilon_c = E_g + \frac{m_r}{m_e}(\hbar\omega - E_g) \tag{S18}$$

$$\epsilon_v = -\frac{m_r}{m_h}(\hbar\omega - E_g) \tag{S19}$$

Solving for μ_h and μ_e is a lot more challenging and this can only be done analytically with additional assumptions, or numerically as shown in SI D.

To make this problem more insightful we introduce a parameter r that tunes how the excess energy $\Delta\mu - E_g$ is distributed over the quasi-Fermi levels μ_e and μ_h, which are given by expressions (S20) and (S21) (a similar approach was introduced by Chen et al.).

$$\mu_e = E_g + r(\Delta\mu - E_g) \tag{S20}$$

$$\mu_h = -(1-r)(\Delta\mu - E_g) \tag{S21}$$

The parameter r is defined such that a value of $r = 0.5$ gives a symmetric quasi-Fermi level splitting between the CB and VB while at a value of $r = 1.0$, μ_h is at the valence band edge and $\mu_e = \Delta\mu$. Combining expressions (S18) and (S19) with Eqs. (S20) and (S21) and substituting them into Eq. (S17) yields Eq. (S22) for the full occupation correction term.

$$[f_v - f_c] = \frac{1}{\exp\left(\frac{-\frac{m_r}{m_h}(\hbar\omega - E_g) + (1-r)(\Delta\mu - E_g)}{k_B T}\right) + 1} - \frac{1}{\exp\left(\frac{\frac{m_r}{m_e}(\hbar\omega - E_g) - r(\Delta\mu - E_g)}{k_B T}\right) + 1} \tag{S22}$$

Although Eq. (S22) is generally valid, a strategy to appropriately tune r should still be found. For intrinsic materials this problem can be approached in several ways, for instance Chen[4] et al. used an expression based on the idea that r can be calculated exactly for an intrinsic material at 0 K. For this approach we note that for an intrinsic semiconductor the density of excited electrons $\Delta n = n$ equals the density of excited holes $\Delta p = p = n$, independent of the excitation conditions. The number of electrons (holes) can be calculated by integrating over the product of the density of states of the conduction band (valence

band) given by[14]

$$\rho_{c/v}(\epsilon) = \frac{1}{2\pi^2}\left(\frac{2m_{e/h}}{\hbar^2}\right)^{\frac{3}{2}} \begin{cases} \sqrt{\epsilon - E_g} \cdot H(\epsilon - E_g) \\ \sqrt{-\epsilon} \cdot H(-\epsilon) \end{cases} \tag{S23}$$

in which $m_{e/h}$ is the effective electron/hole mass of the respective band and the Fermi-Dirac distribution for the electrons (holes), which yields Eq. (S24).

$$\begin{gathered} n = p \\ \int_{-\infty}^{\infty} \rho_c(\epsilon) \cdot f(\epsilon, \mu_e) \cdot d\epsilon = \int_{-\infty}^{\infty} \rho_v(\epsilon) \cdot f(\epsilon, \mu_h) \cdot d\epsilon \end{gathered} \tag{S24}$$

Using the parabolic band approximation and the definition of the Fermi-Dirac distribution for Eq. (S24) we find that it can only be evaluated analytically at 0 K such that the Fermi-Dirac distributions are simple step functions. Employing this approach, Eq. (S24) can be rewritten as (S25).

$$\int_{E_g}^{\mu_e} \frac{1}{2\pi^2} \cdot \left(\frac{2m_e}{\hbar^2}\right)^{\frac{3}{2}} \cdot \sqrt{\epsilon - E_g} \cdot d\epsilon = \int_{\mu_h}^{0} \frac{1}{2\pi^2} \cdot \left(\frac{2m_h}{\hbar^2}\right)^{\frac{3}{2}} \cdot \sqrt{-\epsilon} \cdot d\epsilon \tag{S25}$$

Eq. (S25) is easily simplified by evaluating the integrals, additionally μ_e and μ_h can be expressed in terms of r with Eqs. (S20) and (S21) and we find that $r = \frac{m_r}{m_e}$. By combining this result with expression (S16) an exact solution for the occupation correction term is found given by Eq. (S26). The resulting value of $r = \frac{m_r}{m_e}$ is a reasonable approximation for intrinsic semiconductors at low electron temperatures.

$$[f_v - f_c] = \frac{1}{\exp\left(\frac{-\frac{m_r}{m_h}(\hbar\omega - \Delta\mu)}{k_B T}\right) + 1} - \frac{1}{\exp\left(\frac{\frac{m_r}{m_e}(\hbar\omega - \Delta\mu)}{k_B T}\right) + 1} \tag{S26}$$

Alternatively, Katahara[2] et al. suggested a case where the effective masses of the valence

and conduction band are similar (i.e. $m_h \approx m_e$) while the semiconductor is intrinsic. Now the splitting of the quasi-Fermi-levels happens symmetrically around the center of the band gap, therefore a value of $r = 0.5$ can be assumed and the general expression for $[f_l - f_u]$ given by (S26) reduces to Eq. (S27). Note that due to the symmetry the expression is valid for all temperatures.

$$[f_v - f_c] = 1 - \frac{2}{\exp\left(\frac{\hbar\omega - \Delta\mu}{2k_BT}\right) + 1} \tag{S27}$$

However, both approaches do not take into account doping effects. In fact, for a doped semiconductor, no satisfactory solution for the value of r is proposed yet.

D Numerical calculation of the quasi-Fermi levels and occupation correction

In this section we will look for a way to numerically determine the quasi-Fermi levels μ_e and μ_h as function of the total quasi Fermi-level splitting $\Delta\mu$ and the doping density n_0 such that expression (S17) can be evaluated. As a starting point, we consider the density of states for the parabolic bands given by Eq. (S23). We define the effective masses of the respective bands using

$$m_{e/h} = (m_{\Gamma-K} \cdot m_{\Gamma-A} \cdot m_{\Gamma-M})^{\frac{1}{3}} \tag{S28}$$

to average over the different $\boldsymbol{k}$-directions[16] to follow the isotropic band description in Eqs. (S8) and (S9). Using the published values for hex-Ge based on DFT calculations[17] employing the HSE06 functional to simulate the quasiparticle effect, we find an averaged effective mass of m_e =0.18 for the conduction band and m_h =0.11 for the valence band in units of the free electron mass.

To obtain the electron concentration, Eq. (S23) is first convoluted with a peak function $U(E, T, \mu_e)$ (in complete analogy with the description of the JDOS in SI B) to account for doping induced tail states, then multiplied with the electron Fermi-Dirac function and

integrated resulting in Eq. (S29). To describe the number of holes in the valence band, a similar calculation can be performed resulting in Eq. (S30).

$$n = \Delta n + n_0 = \int_{-\infty}^{\infty} \rho_c(\epsilon) * U(\epsilon, \gamma_c) \cdot f(\epsilon, T, \mu_e) \cdot d\epsilon \tag{S29}$$

$$p = \Delta p = \Delta n = \int_{-\infty}^{\infty} \rho_v(\epsilon) * U(\epsilon, \gamma_v) \cdot [1 - f(\epsilon, T, \mu_h)] \cdot d\epsilon \tag{S30}$$

Note that the broadening parameters $\gamma_{c/v}$ can separately be taken into account for the valence band and conduction band. Unfortunately, the integrals in Eqs. (S29) and (S30) cannot be calculated analytically. We therefore choose to evaluate them, using a numerical trapezoidal integration method. Although these relations can only be solved numerically, they do provide a direct, one-to-one relation between Δn and the quasi-Fermi-levels μ_e and μ_h (and therefore also $\Delta\mu$). By reformulating (S29) and (S30) as a root-finding problem, the quasi-Fermi-levels are determined using Brent's method available in the *root_scalar* function of the *python* package *SciPy*.

$$\mu_e = F_{Brent}(\Delta n, n_0, \gamma_c, T) \tag{S31}$$

$$\mu_h = F_{Brent}(\Delta n, \gamma_v, T) \tag{S32}$$

$$\Delta\mu = \mu_e - \mu_h = F_{Brent}(\Delta n, n_0, \gamma_c, \gamma_v, T) \tag{S33}$$

Eqs. (S31) to (S33) form a framework to directly investigate the behavior of the quasi-Fermi-levels as a function of the minority carrier density for different temperatures and for varying values for γ_c and γ_v. We assume that $\gamma_{JDOS} = \gamma_c = \gamma$ and $\gamma_v = 0$.

The behaviour of both μ_h and μ_e (and therefore $\Delta\mu$) are calculated at different doping densities n_0 and excited charge carrier densities Δn and compared to experimental results. From APT measurements, published in Extended Data Fig. 6 from Fadaly[18] et al., we know that the n-type doping n_0 is of the order of $10^{19}\,\text{cm}^{-3}$ and therefore much higher than the

p-type doping. Therefore, the total number of free electrons n equals the sum of the pumping induced charge carrier density Δn and n_0. Because in a PL experiment the number of excited electrons simply equals the number of excited holes, and because the p-type doping can be neglected the total hole density equals $p = \Delta n$.

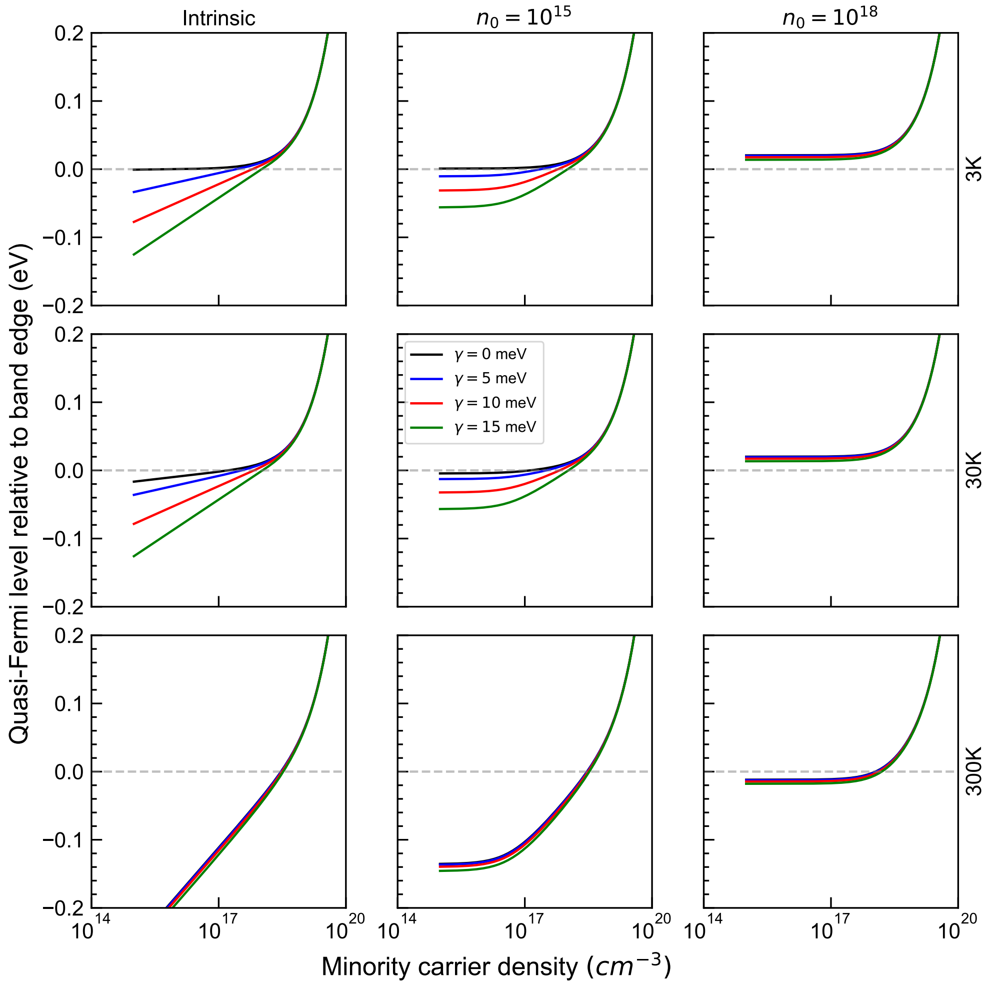

Figure S3: The evolution of the quasi-Fermi level for different temperatures, doping densities and Urbach broadening γ as a function of the minority carrier density. The grey dashed line indicates the band edge at zero energy. A quasi-Fermi level positioned in the band is indicated by a positive relative energy while negative energies indicate the quasi-Fermi level exists in the bandgap.

We illustrate the discussed behaviour of the quasi-Fermi level μ_e as a function of increasing minority carrier density Δp in Fig. S3 for a temperature of 4 K, 40 K and 400 K and for intrinsic or $1 \times 10^{15}\,\mathrm{cm}^{-3}$ or $1 \times 10^{18}\,\mathrm{cm}^{-3}$ of doping. We see that μ_e stays constant in

the low injection regime irrespective of temperature, and only starts to increase when the injection becomes comparable to the doping level. To study the effect of additional states near the band edge, γ has been varied from 0 meV (blue line) to 15 meV (red line). In the case of no broadening and no/low doping at 4 K ($\gamma = 0\,\text{meV}$), μ is independent of Δp and overlaps with the valence band edge. With an increasing broadening parameter γ more states in the band gap become available and μ shifts into the band gap for low minority carrier densities. When Δp is increased, μ shifts back to the band edge showing a linear behavior as function of $\log(\Delta p)$. At increasing carrier temperature the slope of μ versus $\log(\Delta p)$ increases due to a broadened Fermi-Dirac distribution.

E Matrix elements and α_0

The absorption coefficient in semiconductors can be related to the optical matrix element directly.[9–11] This is shown in Eqs. (S6-S13) where assuming $f_v = f_c = 1$, the effective masses, the refractive index and the matrix element are (virtually) constant close to Γ, the absorption coefficient can be split into a constant prefactor multiplied by the energy dependence

$$\alpha(\hbar\omega) = \alpha_0 \frac{\sqrt{\hbar\omega - E_g}}{\hbar\omega} \tag{S34}$$

where the absorption strength scales with the square of the matrix element

$$\alpha_0 \propto |\hat{e} \cdot \boldsymbol{p}_{cv}|^2 \tag{S35}$$

For Zinc-Blende (ZB) or cubic semiconductors the directionally averaged matrix element near the Γ point can be simply expressed in terms of the Kane energy by

$$|\hat{e} \cdot \boldsymbol{p}_{cv}|^2 = \sum_{\sigma=\uparrow,\downarrow} \frac{1}{3} P^2 = \sum_{\sigma=\uparrow,\downarrow} \frac{m_0}{6} E_k = \frac{m_0}{3} E_k \tag{S36}$$

where we used the definition $P^2 = \frac{m_0}{2} E_k$. For an isotropic crystal, which is the case for

ZB semiconductors, the factor $\frac{1}{3}$ accounts for the misalignment of $\boldsymbol{p}_{cv}$ and $\boldsymbol{k}$. The factor 2 accounts for the spin-up and spin-down components. For anisotropic crystals like Wurtzite (WZ) or hexagonal semiconductors, this analysis is less straightforward as $\boldsymbol{k}$ can no longer be assumed along an arbitrary z direction. In this case, the matrix element must be split into a parallel component along the c-axis (∥) and a transverse component (⊥) perpendicular to the c-axis[19,20] which are then combined by averaging over the three polarizations $(2 \times TE + TM)/3$ resulting in[19]

$$|\boldsymbol{p}_{cv}|^2 = \frac{2\,|\boldsymbol{p}_{cv}|_\perp^2 + |\boldsymbol{p}_{cv}|_\parallel^2}{3} = \frac{2}{3}|\boldsymbol{p}_{cv}|_\perp^2 \tag{S37}$$

using the fact that $|\hat{e}\cdot\boldsymbol{p}_{cv}|_\parallel^2 = 0$ as in WZ crystals the parallel transition at $\boldsymbol{k} = 0$ is symmetry forbidden.[21] We can then substitute the relation between the Kane energy and the matrix elements determined by Chuang and Litvinov[19–21] for WZ materials $|\hat{e}\cdot\boldsymbol{p}_{cv}|_\perp^2 = \sum_{\sigma=\uparrow,\downarrow} \frac{m_0}{4} E_{k_\perp}$. This results in

$$|\hat{e}\cdot\boldsymbol{p}_{cv}|^2 = \frac{2}{3}\sum_{\sigma=\uparrow,\downarrow}\frac{m_0}{4}E_{k_\perp} = \frac{2}{3}\left(\frac{m_0}{2}E_{k_\perp}\right) = \frac{m_0}{3}E_{k_\perp} \tag{S38}$$

which is, coincidentally, the same as Eq. (S36) except now only the perpendicular component of the Kane Energy is relevant for the optical transition strength.

Combining this result with Eq. (S11) and the prefactor from Eq. (S12), a direct expression is found relating the absorption strength to the Kane energy for a WZ semiconductor

$$\alpha_0 = \frac{\Lambda m_0}{6\pi^2}\left(\frac{2m_r^*}{\hbar^2}\right)^{\frac{3}{2}} E_{k_\perp} \tag{S39}$$

which together with the characteristic length scale d from the absorptivity forms the $\alpha_0 d$ fitting constant. This expression is identical to the ZB case except that only the ⊥ component of the optical matrix element is used.

Apart from determining the optical transition strength from the simulation result, we also use this derivation to determine reasonable bounds for the $\alpha_0 d$ fitting parameter. For

this purpose, we calculate α_0 both from the Kane energies as calculated with DFT[17] of the (pseudo-direct) weak transition as an indication of the lower bound, as well as from the $\boldsymbol{k} \cdot \boldsymbol{p}$ method for semiconductors[11,19] as an upper bound. We emphasize that the $\boldsymbol{k} \cdot \boldsymbol{p}$ method is only valid for semiconductors with allowed transitions and a conduction band of pure s orbital character at $\boldsymbol{k} = 0$. Although this method is clearly not valid for hex-SiGe, it is still useful to provide an upper bound. In hex-Ge, the Γ_{8c}^{-} conduction band is sp-hybridized and therefore only of partial s character, therefore estimating the matrix elements assuming a pure s conduction band should give a good upper bound of the transition strength. We stress that the equations below are not required to calculate the matrix elements from our results, and are only used for estimations of the parameter space. A first approximation[11,19] of the Kane energy is derived from just the band-gap energy and the effective mass of the conduction band electrons

$$E_k = E_g \left(\frac{m_0}{m_e} - 1 \right) \tag{S40}$$

This equation is commonly extended to include spin-orbit (SO) coupling[9] by

$$E_k = E_g \left(\frac{m_0}{m_e} - 1 \right) \left(\frac{E_g + \Delta_{SO}}{E_g + \frac{2}{3}\Delta_{SO}} \right) \tag{S41}$$

increasing the accuracy of the prediction by including the energy difference from the split-off band to the degenerate light- and heavy-hole bands. For WZ semiconductors, the anisotropy of the crystal requires the addition of an energy splitting between the states composed of p_x, p_y orbitals and p_z orbitals known as the Crystal-Field (CF) splitting, which splits the crystal-hole (CH) valence band from the LH and HH bands. Additionally, the SO coupling then further splits the LH and HH valence bands resulting in three non-degenerate valence bands.[9] Again, it is necessary to separately address the parallel and perpendicular components. To keep notation similar with literature, the variables are changed according

to

$$\begin{aligned} \Delta_1 = \Delta_{CF},\ \Delta_2 = \Delta_3 = \frac{1}{3}\Delta_{SO} \\ \Delta_{SO} = \frac{1}{3}\left(2\Delta_{SO}^{\perp} + \Delta_{SO}^{\parallel}\right) \end{aligned} \tag{S42}$$

and the resulting Kane energies for the two crystal directions can now be written as[19,21]

$$E_{k_\parallel} = E_g\left(\frac{m_0}{m_e^{\parallel}} - 1\right)\frac{(E_g + \Delta_1 + \Delta_2)(E_g + 2\Delta_2) - 2\Delta_3^2}{E_g\,(E_g + 2\Delta_2)} \tag{S43}$$

$$E_{k_\perp} = E_g\left(\frac{m_0}{m_e^{\perp}} - 1\right)\frac{(E_g + \Delta_1 + \Delta_2)(E_g + 2\Delta_2) - 2\Delta_3^2}{(E_g + \Delta_1 + \Delta_2)(E_g + \Delta_2) - \Delta_3^2} \tag{S44}$$

Since the Kane model underestimates the matrix element by ≈17 % for ZB materials, a similar underestimate is expected for WZ.[19]

Table S1: Material parameters used to determine the Kane energy and absorption coefficient for hex-Ge as calculated by DFT including QP corrections.[17,22] For the hex-Ge bandgap, the value determined from photoluminescence experiments is used.[18]

Parameter (DFT)	Value
$\Delta_1 = \Delta_{CF}$	0.288 eV
$\Delta_{SO}^{\parallel}$	0.329 eV
$\Delta_{SO}^{\perp}$	0.320 eV
$m_e^{\parallel}/m_0$	0.997
$m_e^{\perp}/m_0$	0.076
$m_h^{\parallel}/m_0$	0.463
$m_h^{\perp}/m_0$	0.055
n_r	3.75
**Parameter (Exp.)**	**Value**
E_g	0.360 eV

Using the parameters from Table S1, we calculate that $E_{k_\perp} \approx 5.27$ eV and $E_{k_\parallel} \approx 2.15$ meV in qualitative agreement with group theory at the BZ center. Although the value of $E_{k_\perp}$ is a little lower than for III-V semiconductors (≈25 eV ZB GaAs/InAs/InP) and (13.9 eV-15.7 eV for GaN),[21] the smaller value of $E_{k_\perp}$ for hex-Ge is mostly due its relatively tiny bandgap. Notably, the value of the Kane energy $E_{k_\perp}$ estimated from Eq. (S43) is three orders of magnitude higher than the $E_{k_\perp} \approx 2$ meV calculated by DFT[17] for ideal bulk 2H-Ge. Due

to the nanowire geometry of our hex-Ge nanowires, the effective medium approximation is most useful to estimate the refractive index. The effective refractive index of the nanowire layer is between the refractive index of the hex-Ge semiconductor and that of air. As a worst case scenario, we assume $n_r \approx 1$ and $d \approx 6.4\,\mu\text{m}$. Combining insights with Eq. (S39) allows us to estimate reasonable bounds for the fitting parameter $\alpha_0 d$ which are estimated to be $\approx 0.012\,\text{eV}^{1/2}$ for the lower bound and $\approx 30.3\,\text{eV}^{1/2}$ for the upper bound. During the actual optimization procedure these bounds are further relaxed (down/up respectively) to allow for variation outside of this initial estimate.

F Stability of the particle swarm optimization procedure.

Since the particle swarm optimization is a heuristic method, we assess the robustness of the obtained solution by analyzing the PSO datasets. In Fig. S4, we present a comprehensive overview of the PSO parameter space and the corresponding fitness functions. All parameters and the fitness functions are shown on a logscale for clarity. In addition, the fitting parameters and the fitness functions are plotted on a zoomed-in linear scale in Fig. S5. On the diagonal panels of Fig. S4, we show for each parameter the overall minimum fitness over 100 bins of the parameters' fitting bounds. The maximum fitness found on the diagonal ≈ 200 is taken as the upper limit of the color bar for the off-diagonal panels in Fig. S4. The diagonal panels in Fig. S4 clearly show that a global minimum of the fitness function is found for each fitting parameter. The realization of a global minimum of the fitness function is even more clearly visible in the diagonal panels of Fig. S5. On the off-diagonals panels, we show the cross-correlation stability for each of the four parameters fitted with the PSO approach, ζ, $\alpha_0 d$, n_0 and the $\Delta p/G$. This is done by taking 100 bins of both parameters' fitting bounds and calculating overall minimum fitness for each 2D pixel. A strong correlation between the parameters would then show as a line of (nearly) equal fitness. Fortunately, all plots show a clear global minimum, highlighted with crossing red lines, indicating that the solution is robust.

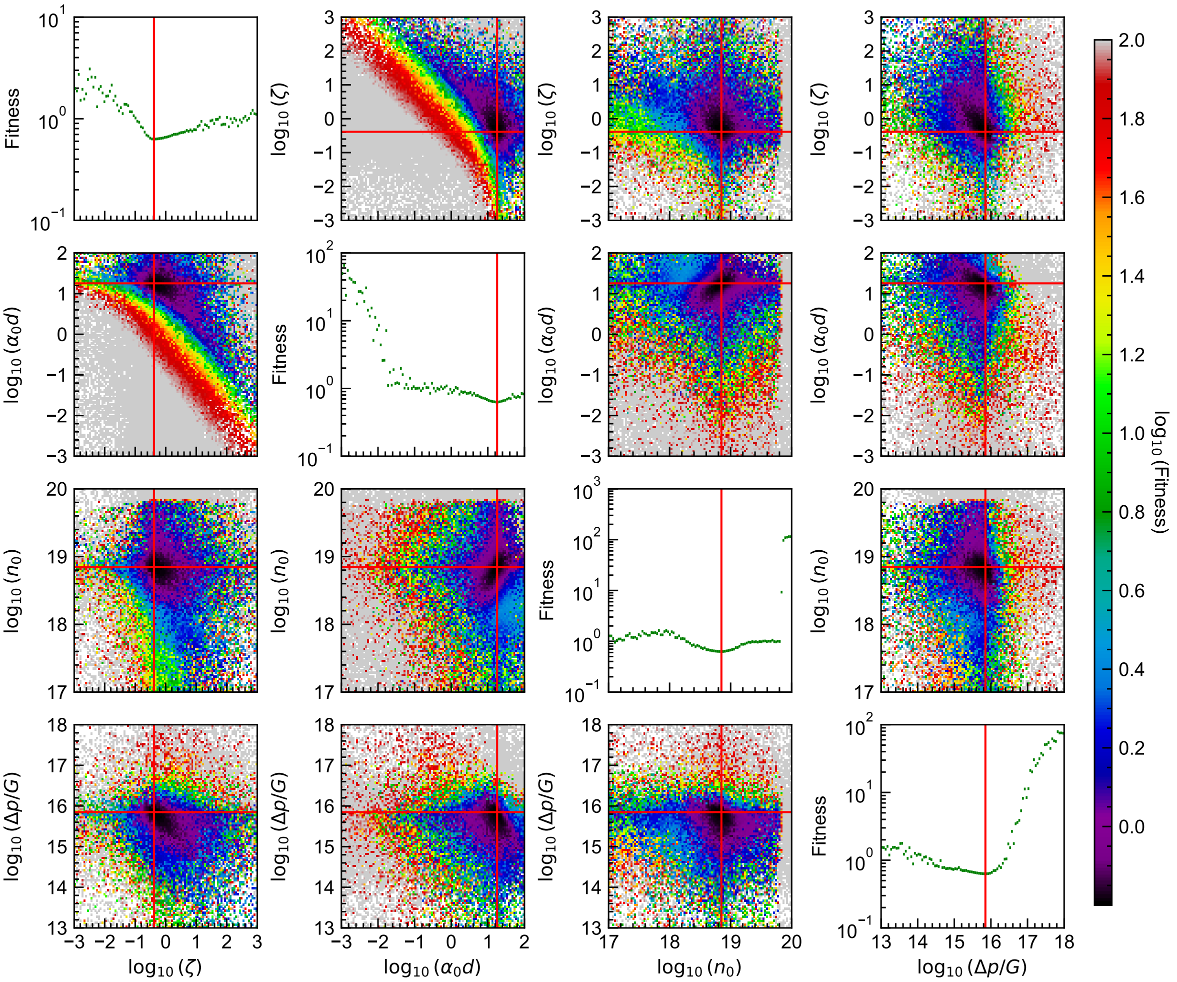

Figure S4: Statistics showing the stability of the PSO model as well as potential cross correlations between the fitting parameters on a logarithmic scale. The diagonal panels show the overall minimum values of the fitness function per bin for 100 bins over the fitting range for each different fitting parameter, while the off-diagonal panels show the cross-correlation of the minimum fitness function between the different pairs of parameters for each pixel of 100 bins for each parameter. The red lines indicate the best solution of each respective parameter. The colorbar shows the range of the fitness function, starting at the minimum fitness level, up to the maximum fitness level observed on the diagonal

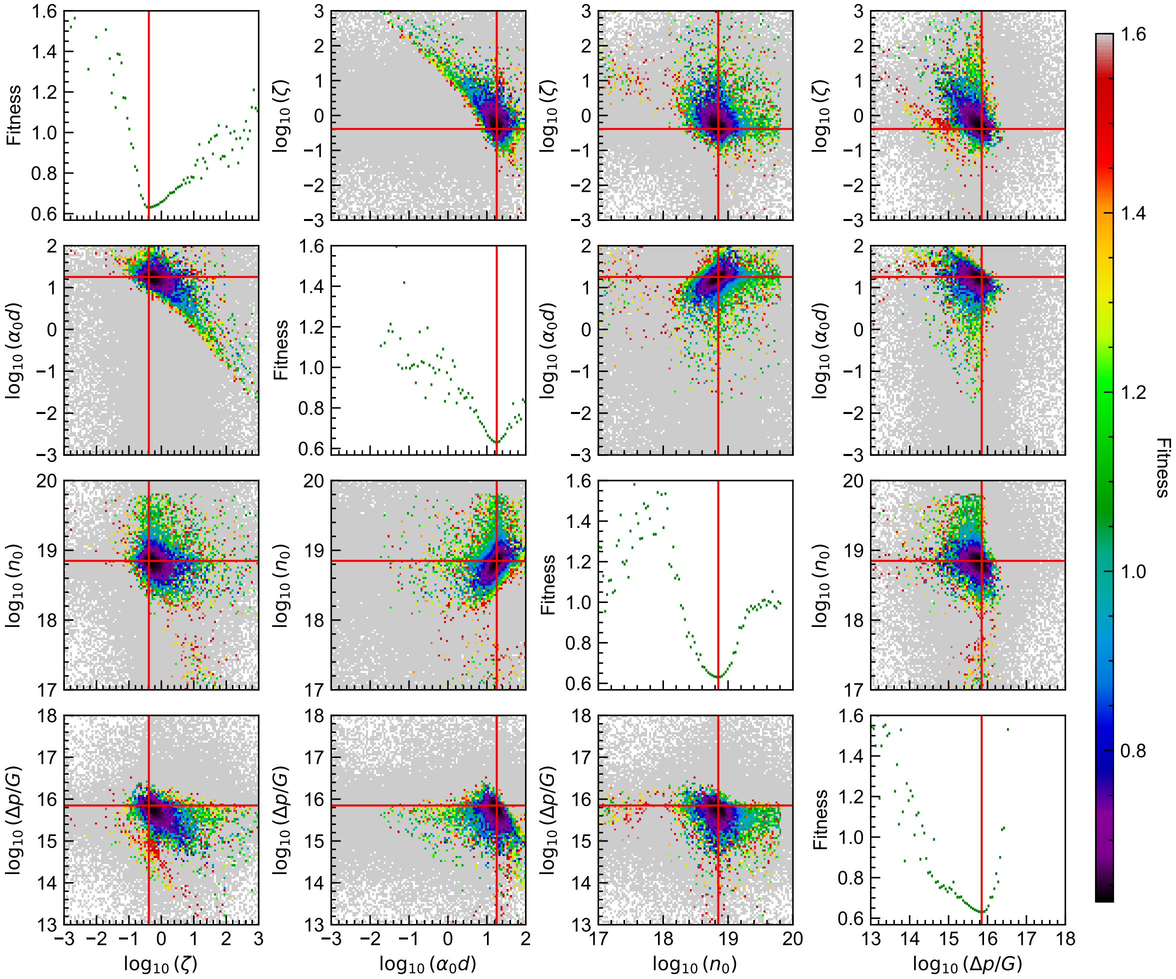

Figure S5: Statistics showing the stability of the model and potential cross correlations between the fitting parameters on a linear scale. The diagonal panels show the overall minimum value fitness function per bin for 100 bins over the fitting range for each fitting parameter, while the off-diagonals show the cross-correlation of the minimum fitness function between the different pairs of parameters for each pixel of 100 bins for each parameter. The red lines indicate the best solution of each respective parameter. The colorbar shows the range of the fitness function.

G Estimation of the scaling factor ζ

To estimate the magnitude of the fitted scaling factor ζ from the experiment, we start with the prefactor of the LSW model $(4\pi^2\hbar^3c^2)^{-1}$ =$9.88 \times 10^{26}\,\mathrm{m^{-2}\,eV^{-3}\,s^{-1}}$. The diameter of our spotsize on the sample is estimated to be 45 µm. The spectral resolution of our FTIR spectrometer at $32\,\mathrm{cm^{-1}}$ corresponds to a binsize $\delta_E = 3.97\,\mathrm{meV}$. The typical MCT detector sensitivity is specified* to be $R_{det} = 750\,\mathrm{V\,W^{-1}}$. Around a photon energy of 1 eV, this corresponds to $750\,\mathrm{V\,A^{-1}}$. With a pre-amplifier gain of $g = 80$, we find a prefactor of $\frac{gR_{det}\delta_E A}{4\pi^2\hbar^3c^2}$≈$60\,\mathrm{V\,eV^{-2}}$ without considering the PL collection efficiency or mirror/window losses. We estimate the window (Sapphire, 3x CaF_2, CdTe) and mirror losses (9x Au) to add a total transmission of 32.8 %. For the losses concerning the NA of the parabolic mirror which is focussing the light on the hex-Ge sample, we assume a loss of ∼50 %, which is likely slightly underestimated. Finally, the resulting estimation for the scaling variable is $\zeta \approx 9.8\,\mathrm{V\,eV^{-2}} \sim 10^1\,\mathrm{V\,eV^{-2}}$. Since this is a very rough estimate, we choose a fitting range of $10^{-3}\,\mathrm{V\,eV^{-2}}$ to $10^3\,\mathrm{V\,eV^{-2}}$.

H External radiative efficiency of hex-Ge

After optimizing the LSW model by employing the ζ parameter to fit the amplitude, we can calculate the spectra using the original prefactor from Eq. (2) of the main text ($(4\pi^2\hbar^3c^2)^{-1} = 9.88 \times 10^{26}\,\mathrm{m^{-2}\,eV^{-3}\,s^{-1}}$) to get the amplitude in the units of absolute photon spectral flux density. Integrating these spectra over energy then gives the emitted absolute PL flux density of the hex-Ge nanowires. Fortunately calculating the laser flux density is trivial knowing the excitation density and photon energy 1.27 eV. This allows us to visualize the integrated photon flux density as a function of the laser flux density in Fig. S6 on a double logarithmic scale where a vertical translation indicates the multiplication by a constant and with the laser flux density as the limit of unity efficiency. From this relation we find an average External Radiative Efficiency (ERE) of $(57 \pm 7)\,\%$ over three orders of magnitude varying excitation

*https://nicoletcz.cz/app/uploads/2021/07/8d6c9e08.pdf

density. This indicates that the radiative efficiency of hex-Ge is excellent and crystal defects or impurities are not causing significant non-radiative recombination.

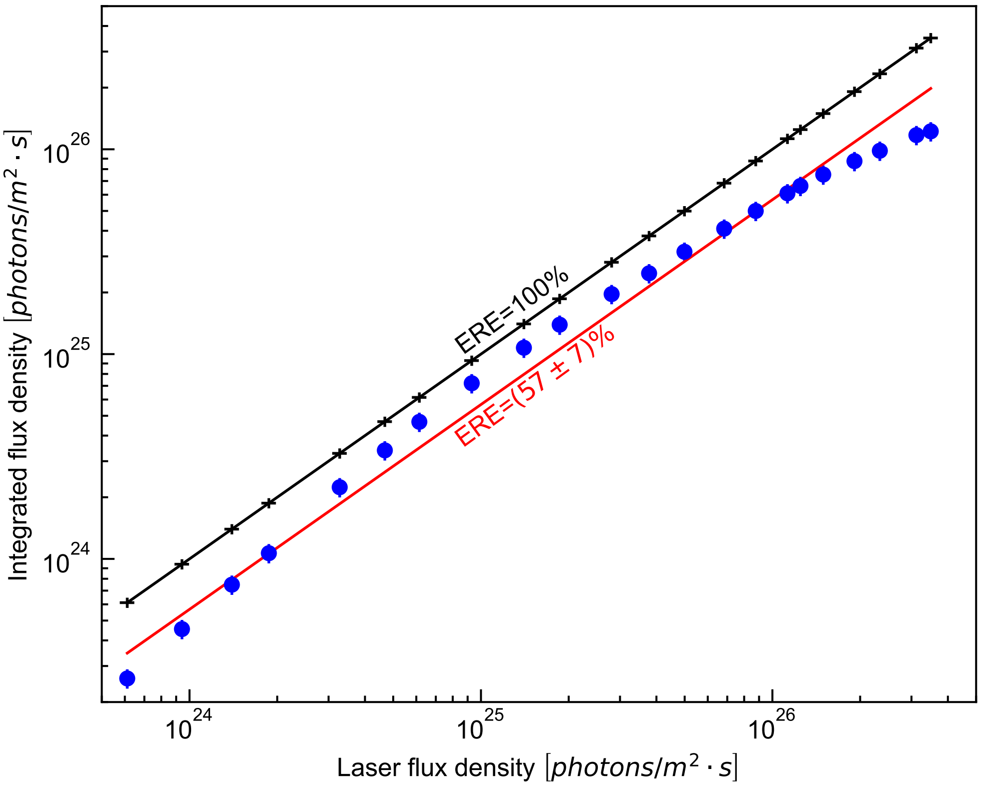

Figure S6: The external radiative efficiency of the integrated flux density calculated using the LSW model as a function of the laser flux density. The upper limit of unity efficiency is shown in black and the average ERE, due to the double logarithmic scale, is determined from the offset of the linear fit.

I Sample geometry

To calculate the lifetime from the g/G_{laser} parameter we need knowledge of the sample geometry. The geometry for the hex-Ge sample is given in Table S2, where all distances are determined from scanning electron microscopy. The GaAs core diameter was determined from cross-sectional HAADF-STEM images on a different sample with thinner shells grown on similar sized cores, we show an example image in Fig. S7 with a partial false coloring indicating the WZ-GaAs core in blue and the hex-Ge shell in red.

Table S2: Geometry of the NW sample determined from SEM images. The NWs are thicker at the top than at the bottom, therefore we model the nanowires as two stacked cylinders.

Length	Value
Center-to-center pitch	(2.00 ± 0.02) µm
Thick total NW diameter	(1.12 ± 0.08) µm
Thin total NW diameter	(0.71 ± 0.03) µm
GaAs core diameter	(175 ± 5) nm
NW length total	(6.4 ± 0.4) µm
NW length thick	(1.5 ± 0.2) µm
NW length thin	(4.8 ± 0.4) µm
NW volume	$(3.28 \pm 0.33)\ \mu m^3$
NW density	$(0.250 \pm 0.005)\ \mu m^{-2}$

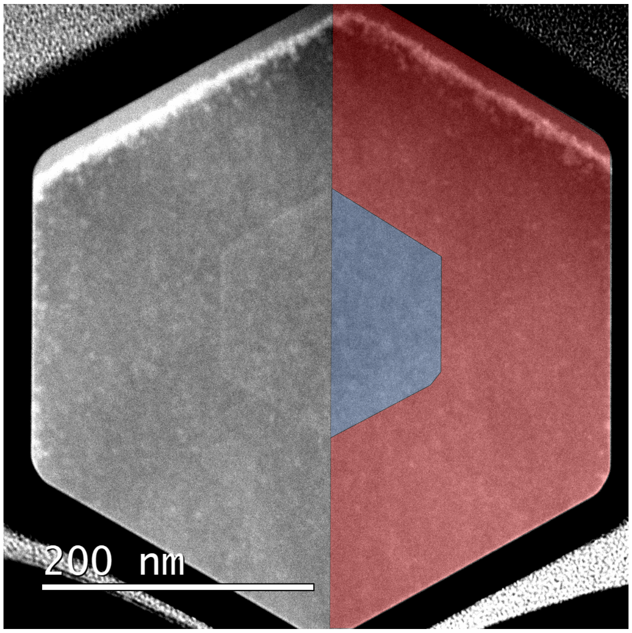

Figure S7: A cross-sectional HAADF-STEM image of a sample grown with thinner shells on similar cores showing the core-shell structure of the sample indicated by the partial false coloring: blue for WZ-GaAs and red for Hex-Ge.

References

(1) Trupke, T.; Daub, E.; Würfel, P. Absorptivity of silicon solar cells obtained from luminescence. *Solar Energy Materials and Solar Cells* **1998**, *53*, 103–114.

(2) Katahara, J. K.; Hillhouse, H. W. Quasi-Fermi level splitting and sub-bandgap absorptivity from semiconductor photoluminescence. *Journal of Applied Physics* **2014**, *116*, 173504.

(3) Daub, E.; Würfel, P. Ultralow Values of the Absorption Coefficient of Si Obtained from Luminescence. *Physical Review Letters* **1995**, *74*, 1020–1023.

(4) Chen, H.-L.; Himwas, C.; Scaccabarozzi, A.; Rale, P.; Oehler, F.; Lemaître, A.; Lombez, L.; Guillemoles, J.-F.; Tchernycheva, M.; Harmand, J.-C.; Cattoni, A.; Collin, S. Determination of n-Type Doping Level in Single GaAs Nanowires by Cathodoluminescence. *Nano Letters* **2017**, *17*, 6667–6675.

(5) Chen, H.-L.; De Lépinau, R.; Scaccabarozzi, A.; Oehler, F.; Harmand, J.-C.; Cattoni, A.; Collin, S. Quantitative Assessment of Carrier Density by Cathodoluminescence. II. GaAs Nanowires. *Physical Review Applied* **2021**, *15*, 024007.

(6) Chen, H.-L.; Scaccabarozzi, A.; De Lépinau, R.; Oehler, F.; Lemaître, A.; Harmand, J.-C.; Cattoni, A.; Collin, S. Quantitative Assessment of Carrier Density by Cathodoluminescence. I. GaAs Thin Films and Modeling. *Physical Review Applied* **2021**, *15*, 024006.

(7) Cabrera, C.; Contreras-Solorio, D.; Hernández, L. Joint density of states in low dimensional semiconductors. *Physica E: Low-dimensional Systems and Nanostructures* **2016**, *76*, 103–108.

(8) Bastard, G.; Delalande, C.; Guldner, Y.; Voisin, P. *Advances in Electronics and Electron Physics*; Academic Press, 1988; Vol. 72; pp 1–180.

(9) Vurgaftman, I.; Lumb, M. P.; Meyer, J. R. *Bands and Photons in III-V Semiconductor Quantum Structures*; Oxford University Press, 2020; pp 93–138.

(10) Chuang, S. L. *Physics of photonic devices*, 2nd ed.; Wiley series in pure and applied optics; John Wiley & Sons: Hoboken, N.J., 2009.

(11) Yu, P. Y.; Cardona, M. *Fundamentals of Semiconductors*; Graduate Texts in Physics; Springer Berlin Heidelberg: Berlin, Heidelberg, 2010.

(12) Pelant, I.; Valenta, J. *Analytical and Bioanalytical Chemistry*; Oxford University Press, 2012; Vol. 409; pp 1473–1474.

(13) Grundmann, M. *The Physics of Semiconductors*; Graduate Texts in Physics; Springer International Publishing: Cham, 2016.

(14) Klingshirn, C. F. *Semiconductor Optics*; Graduate Texts in Physics; Springer Berlin Heidelberg: Berlin, Heidelberg, 2012.

(15) Urbach, F. The Long-Wavelength Edge of Photographic Sensitivity and of the Electronic Absorption of Solids. *Physical Review* **1953**, *92*, 1324–1324.

(16) Green, M. A. Intrinsic concentration, effective densities of states, and effective mass in silicon. *Journal of Applied Physics* **1990**, *67*, 2944–2954.

(17) Rödl, C.; Furthmüller, J.; Suckert, J. R.; Armuzza, V.; Bechstedt, F.; Botti, S. Accurate electronic and optical properties of hexagonal germanium for optoelectronic applications. *Physical Review Materials* **2019**, *3*, 034602.

(18) Fadaly, E. M. T. et al. Direct-bandgap emission from hexagonal Ge and SiGe alloys. *Nature* **2020**, *580*, 205–209.

(19) Chuang, S. Optical gain of strained wurtzite GaN quantum-well lasers. *IEEE Journal of Quantum Electronics* **1996**, *32*, 1791–1800.

(20) Litvinov, V. I. Optical transitions and gain in group-III nitride quantum wells. *Journal of Applied Physics* **2000**, *88*, 5814–5820.

(21) Chuang, S. L.; Chang, C. S. k·p method for strained wurtzite semiconductors. *Physical Review B* **1996**, *54*, 2491–2504.

(22) Borlido, P.; Bechstedt, F.; Botti, S.; Rödl, C. Ensemble averages of ab initio optical, transport, and thermoelectric properties of hexagonal Si x Ge 1 - x alloys. *Physical Review Materials* **2023**, *7*, 014602.
